# Oceanographic setting influences the prokaryotic community and metabolome in deep-sea sponges

**DOI:** 10.1038/s41598-022-07292-3

**Published:** 2022-03-01

**Authors:** Karin Steffen, Anak Agung Gede Indraningrat, Ida Erngren, Jakob Haglöf, Leontine E. Becking, Hauke Smidt, Igor Yashayaev, Ellen Kenchington, Curt Pettersson, Paco Cárdenas, Detmer Sipkema

**Affiliations:** 1 Pharmacognosy, Department of Pharmaceutical Biosciences, Uppsala University, Husargatan 3, 751 24 Uppsala, Sweden; 2 Department of Microbiology and Parasitology, Faculty of Medicine and Health Sciences, Warmadewa University, Jln Terompong 24, Denpasar, Bali 80239 Indonesia; 3 Laboratory of Microbiology, Wageningen University and Research, Stippeneng 4, 6708 WE Wageningen, The Netherlands; 4 Analytical Pharmaceutical Chemistry, Department of Medicinal Chemistry, Uppsala University, Husargatan 3, 751 23 Uppsala, Sweden; 5 Aquaculture & Fisheries, Wageningen University and Research, De Elst 1, 6708 WD Wageningen, The Netherlands; 6 Department of Fisheries and Oceans, Bedford Institute of Oceanography, Dartmouth, B2Y 4A2 Canada

**Keywords:** Metabolomics, Boreal ecology, Microbial ecology, Microbiome

## Abstract

Marine sponges (phylum Porifera) are leading organisms for the discovery of bioactive compounds from nature. Their often rich and species-specific microbiota is hypothesised to be producing many of these compounds. Yet, environmental influences on the sponge-associated microbiota and bioactive compound production remain elusive. Here, we investigated the changes of microbiota and metabolomes in sponges along a depth range of 1232 m. Using 16S rRNA gene amplicon sequencing and untargeted metabolomics, we assessed prokaryotic and chemical diversities in three deep-sea sponge species: *Geodia barretti*, *Stryphnus fortis*, and *Weberella bursa*. Both prokaryotic communities and metabolome varied significantly with depth, which we hypothesized to be the effect of different water masses. Up to 35.5% of microbial ASVs (amplicon sequence variants) showed significant changes with depth while phylum-level composition of host microbiome remained unchanged. The metabolome varied with depth, with relative quantities of known bioactive compounds increasing or decreasing strongly. Other metabolites varying with depth were compatible solutes regulating osmolarity of the cells. Correlations between prokaryotic community and the bioactive compounds in *G. barretti* suggested members of Acidobacteria, Proteobacteria, Chloroflexi, or an unclassified prokaryote as potential producers.

## Introduction

Sponges (phylum Porifera) are sessile filter-feeding animals found worldwide in shallow and deep, marine and freshwater habitats. Sponges host an exceptionally rich and diverse associated microbiota, together referred to as a ‘holobionts’^1^, and are frequently grouped into high and low microbial abundance sponges (HMA and LMA sponges). Besides common marine prokaryotes, a considerable part of the prokaryotic communities within sponges is affiliated with so-called ‘sponge-enriched clusters’ indicating their adaptation to the sponge hosts^2^. These sponge-associated prokaryotic communities are acquired both through horizontal and vertical transmission of prokaryotic symbionts^1,3,4^ though recent work has challenged both the specificity of sponge-associated prokaryotes^5^ as well as the fidelity of their vertical inheritance^6^. Indeed, despite a high number of microbiome studies and their links to various temporal, spatial or environmental aspects, factors governing assembly and variation of the prokaryotic communities are still a matter of debate. As work on sponge prokaryotic community composition is usually based on shallow-water specimens^7^, this lack of understanding is particularly apparent in the deep-sea, where sampling is sparse^8–10^. In sponges from surface waters to 200 m depth, shifts in prokaryotic communities have been attributed to a combination of factors such as nutrient availability via light availability^11–13^. One study of a diverse set of deeper sponges (472–4160 m) found no evidence for the influence of depth on the sponge-associated microbiota^9^. Meanwhile, another study reported sponge-associated clusters of ASVs to vary in the proximity of a seamount (575 –2184 m)^10^. Depth is a proxy for correlated variables that may directly influence the sponge holobionts, that is, water mass with associated physiochemical and nutrient properties, hydrostatic pressure, light, and food supply.

The high diversity of sponge-associated prokaryotes, each producing its own specific set of compounds, has been used to partially explain the exceptional diversity of chemical compounds in sponges^14,15^ and positions them as leading sources for novel marine bioactive compounds^16,17^. However, the extent of the contribution of microbes to the holobiont chemodiversity is still poorly understood and a topic of high interest. To address this connection, studies have targeted specific bacteria or bacterial groups as producers of specific compounds^18–21^. Profiling of (mostly unknown) specialized metabolites with untargeted metabolomics is a less common approach in sponges and so far, it has essentially been used in demosponge chemotaxonomy^22–27^. Only two works connected the microbial community and the metabolome^28,29^, neither of which investigated a deep-sea gradient.

In this study, we addressed (a) whether shifts in prokaryotic community composition extend beyond the euphotic zone, (b) whether changes in depth and/or prokaryotic community composition are reflected in the metabolome and (c) whether we can use these potential variations to link microbes and metabolome and suggest putative producers of bioactive compounds. We address these questions through 16S rRNA gene sequencing and ultra-high performance liquid chromatography high-resolution mass spectrometry (UPLC-HRMS) in three North Atlantic deep-sea demosponge species, *Geodia barretti* Bowerbank, 1858^30^, *Stryphnus fortis* (Vosmaer, 1885)^31^ and *Weberella bursa* Vosmaer, 1885^31^. *G. barretti* is an HMA sponge^32^ and *S. fortis* was hypothesised to be an HMA sponge, too, whereas *W. bursa* was hypothesised to be an LMA sponge (N. Boury-Esnault *pers. comm.*). We collected 20, 15 and 17 specimens respectively, along a depth gradient ranging from 244 to 1476 m.

## Methods

### Sampling

**Figure 1:**
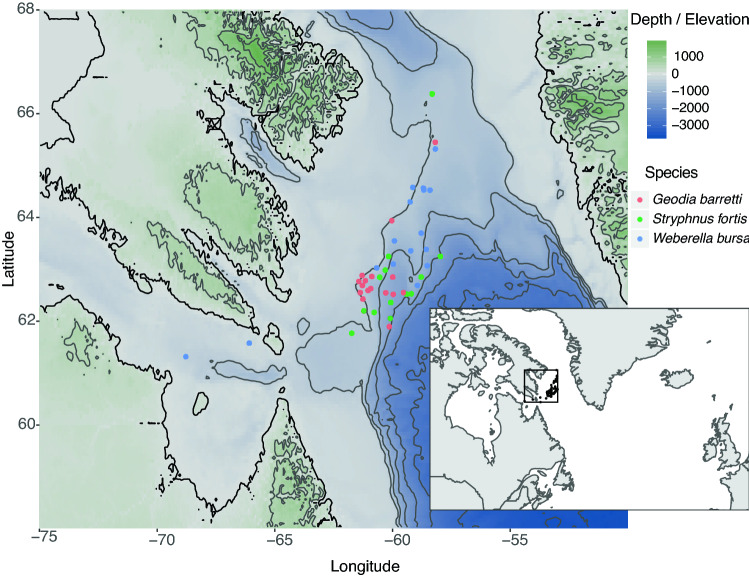
Map of the slope in the Davis Strait in the North Atlantic, where the samples were collected. The insert shows the geographic location of the sampling area (black box) between Canada and Greenland. Samples were collected at depths ranging from 244 to 1476 m. Figure made with R v3.5.1 (https://www.r-project.org) with packages marmap and ggplot2.

Sponge specimens were sampled by the crews of the R/V *Pâmiut* of the Greenland Institute of Natural Resources during cruises conducted by Fisheries and Oceans Canada in the Davis Strait using Alfredo/Cosmos benthic trawls taken between a depth of 244 m and 1476 m (Fig. 1). All samples were taken during the same season (September–October, Autumn) from 2011 to 2015. Samples were frozen on board (− 20 °C) and shipped to the Bedford Institute of Oceanography (BIO, Dartmouth, Nova Scotia, Canada) for identification. Freeze-dried subsamples from the choanosome (i.e. inside of the sponge, to reduce the risks of surface contamination by epibionts) were sent to Uppsala University and stored at 8 °C until processing for global metabolite profiling. Sample identification was reassessed and all specimens of *S. fortis* were checked for the presence of *Hexadella dedritifera*^33^ overgrowth. Frozen subsamples were sent to Wageningen University & Research and stored at − 20 °C until processing for prokaryotic community analyses.

Detailed sample information can be found in Table S1 (deposited at PANGAEA https://doi.pangaea.de/10.1594/PANGAEA.909246).

### Study site, geography and oceanography

The maximal geographic distance across the sampling site was 786 km, covering a depth range of 1232 m. To identify the potential effect of oceanographic conditions (e.g. seawater properties, currents and vertical velocities, water mass origin, age and mixing history) on sponge prokaryotic community compositions and metabolomes, all oceanographic data available for the study area were assembled. These oceanographic observations came from the World Ocean Database (https://www.nodc.noaa.gov/OC5/WOD/pr_wod.html) and Bedford Institute of Oceanography archives, and from Argo float profiles^34^. All vertical profiles of temperature and salinity obtained from discrete (e.g., water samples and reversing thermometers) and initially continuous and later subsampled measurements were checked for errors and vertically interpolated for every 5 m.

### 16S rRNA gene amplicon sequencing and analyses

For each sponge individual a match head of tissue was cut out of the frozen tissue samples with a sterile scalpel and washed three times in artificial seawater to remove any attached debris^35^. DNA extraction and two-step PCR reaction (16S rRNA gene: 515F 5′- GTG YCA GCM GCC GCG GTA A-3′, 806R 5′- GGA CTA CNV GGG TWT CTA AT-3′) were performed following procedures previously described^36^. The barcoded 16S rRNA gene amplicons were sequenced at GATC Biotech AG (Constance, Germany; now part of Eurofins Genomics Germany GmbH) by Illumina Miseq sequencing. Raw data was analysed using NG-Tax (Galaxy version 1.0), for detail see Supplementary methods^37^. Forward and reverse paired-end reads were trimmed to 70 nucleotides and concatenated, resulting in sequences of 140 bp used for subsequent sequence data processing. Taxonomy was assigned with a customized version of the SILVA 128 SSU database^38^, and ASVs (amplicon sequence variants) classified as chloroplasts were removed. Sequencing data was deposited at the NCBI Sequence Read Archive (SRA) with accession number SRP142603 under file numbers SRX3993926–SRX3993970.

### Metabolite extraction, UPLC-HRMS acquisition and analyses

Freeze-dried sponge tissue was ground and 50 mg of the powder was extracted with 4.5 mL 70% methanol/Milli-Q (MQ) water for one hour at room temperature on a shaker at low speed. The vials were centrifuged at 2000 × g for 10 min at 4 °C and 2 mL of the supernatant was dispensed into two glass vials and dried on a heating block (43 °C) under a stream of nitrogen. Dry extracts were kept at − 80 °C until further analyses.

All samples were analysed with UPLC-HRMS, in hydrophilic interaction liquid chromatography (HILIC) and reversed-phase liquid chromatography (RP), in positive and negative ionization mode^39^.

Dried extracts were dissolved in 50 µL MQ water and 175 µL acetonitrile (MeCN) for HILIC, or 140 µL MQ water and 10 µL MeCN for RP analyses. Upon addition of the organic solvent for HILIC samples they separated into two immiscible layers. The vials were centrifuged for 3 min at 2000 × g to achieve complete separation and only the top layer (~ 150 µL) was transferred to an MS-vial for analysis. A 5 µL aliquot from each individual MS-vial for HILIC and RP chromatography, respectively, was combined to produce a quality control (QC) sample. The extracts were analysed on an Acquity I-Class UPLC coupled to a G2S Synapt Q-TOF with an electrospray ionization (ESI) ion source (all Waters Corp., Milford, Massachusetts, USA). Chromatographic separation in HILIC mode was performed on an Acquity UPLC BEH Amide column (1.7 μm, 2.1 mm i.d. × 50 mm, Waters Corp.) and in RP mode on an Acquity UPLC BEH C18 column (1.7 μm, 2.1 mm i.d. × 50 mm, Waters). The flow rate was 0.4 mL/min and the injection volume was 5 µL in all experiments. Data were acquired in MS^E^ mode, and lock mass correction was applied in both positive and negative mode. Prior to each analysis, the instrument was calibrated in the *m/z* range 50 to 1500 and QC injections were made to condition the column and to ensure stable retention times and signal intensities. The study samples were analysed in randomized order with QC injections interspaced every sixth injection.

Raw data from the four UPLC-HRMS experiments were processed separately. Files were converted to CDF files with Databridge/MassLynx (Waters Corp., Milford, Massachusetts, USA), sorted into folders by species and processed with the R packages XCMS^40^ and CAMERA^41^. The output is a table with signal intensity per sample and ‘feature’. Each feature can be annotated as metabolite, adduct, isotope, or lack an annotation. The four resulting feature tables were filtered in R in three different ways (“cleaned”, “pc_group”, “ion”), as follows. Features eluting in the void (< 40 s) and features with a coefficient of variation > 30% in the QC samples were removed (data sets called “cleaned”). Based on these “cleaned” feature tables, adducts and overrepresented features were removed by retaining only the feature with the greatest cumulative signal per CAMERA pc_group (“pc_group”). In addition, based on the “cleaned” feature tables, only features explicitly annotated as [M + H]^+^ in positive ESI mode and [M-H]^−^ in negative ESI mode were retained (“ion”). Metabolomic data is deposited at MetaboLights (www.ebi.ac.uk/metabolights/MTBLS1388) and extended methods are found in the supplementary information.

### Computational and statistical analyses

Data sets (ASV table, features tables, meta data) produced as described above were analysed in R v3.5.1. For the prokaryotes, the effect of different data transformations was evaluated by principal component analysis (PCA) and non-metric multidimensional scaling (NMDS) using Bray–Curtis distance (Fig. S1). PERMANOVA, function ‘adonis’ in R package vegan^42^, was used to test for significant differences in prokaryotic community composition between sponge species. Alpha diversity of the samples was calculated with the R package phyloseq. For each sponge species, ASVs were classified as common if their average relative abundance was > 0.25%. ASVs classified as common were cross-checked for being sponge enriched following a procedure described by Dat et al.^43^. For subsequent ecological analyses, we assessed the rank correlation between dissimilarity indices and gradient separation based on different data transformations and applied the method yielding highest similarity with the Bray–Curtis index. To investigate the effect of depth on the sponge-associated prokaryotes, we fitted all available environmental variables (latitude, longitude, depth, salinity, temperature, sampling year) onto unconstrained ordinations (NMDS with Bray–Curtis distance) of the three species’ prokaryotic communities. In addition, we assessed multicollinearity among the environmental variables and then included only those environmental variables with a variance inflation factor < 10 to build a full constrained model as part for an automated stepwise model building approach evaluated with an ANOVA. The relative abundances of ASVs were correlated (Pearson) to sample depth to infer whether ASVs were ‘increasing’ or ‘decreasing’ with increasing sample depth (*p* ≤ 0.05), and noted as ‘increasing trend’ or ‘decreasing trend’ with increasing sample depth for non-significant correlations (*p* > 0.05). Pairwise sequence similarity was calculated with seqinr and a t-test was performed comparing the relative abundance above and below 1000 m for each ASV. The resulting p-values were corrected for multiple testing (FDR). The phylogeny of the ASVs was inferred with RAxML analyses implemented in CIPRES Science Gateway V. 3.3^44^ and all annotations were mapped on the phylogram in iToL^45^.

To investigate the effect of depth on the holobiont metabolomes, we used orthogonal projection of latent structures (OPLS) models, a supervised ordination method separating predicted variation (depth) from all orthogonal variation (R package ROPLS^46^). OPLS models predicting variation with depth (n = 36) were built for all combinations of experimental setups (n = 4), species (n = 3), and feature filtering (n = 3). Seven-fold cross-validation is performed by the function resulting in a pQ2 value. Models were rejected due to overfitting if they yielded a pQ2 > 0.05. Model fit was evaluated to assess whether depth was affecting the variation of the metabolomes’ features. In an OPLS, all variables (metabolome features) are assigned a “variable influence on projection” (VIP) score: a variable with a VIP score > 1 is considered important to the model. The VIP score is not a statistic test and needs to be supported by such for validity. Known metabolite features from *G. barretti* and *S. fortis*, and the features with the highest VIP scores in the OPLS model of the HILIC positive “cleaned” data set were manually annotated in the metabolome and their signal intensities across the samples were extracted. As the patterns and models based on unidentified metabolomic features underlying the OPLS model should be interpreted with caution, we applied a dual feature identification approach: (1) manually extracting signal intensities of known bioactive compounds and (2) de novo identification of features designated as VIPs by a general OPLS model including all sponge samples combined. Standards were ordered to confirm the identity of VIPs in a repeated UPLC-HRMS/MS experiment, and annotations were confirmed/rejected based on retention time and matching MS/MS spectra. The correlation of the signal intensity and sample depth was tested.

To test overall similarity between the prokaryote and metabolome data sets, we used a Mantel test on the distance matrices (Bray–Curtis for the prokaryotes and Euclidean dissimilarity for the metabolome) and Procrustes rotations.

The microbial interaction network for the *G. barretti*-associated prokaryotes was generated following suggestions by Weiss et al.^47^. ASVs present in less than three samples were removed and the sparsity and N_eff_ of the remaining ASV table was calculated. The ASV table containing 289 remaining ASVs was formatted according to the requirements of each network inference tool. Prokaryotic interaction networks were generated using MENA (using Pearson and Spearman correlation coefficients)^48^ (http://ieg4.rccc.ou.edu/mena), SparCC^49^, fastLSA^50^ and MIC (R package minerva)^51^. The resulting networks were filtered to only retain positive interactions (edges). The consensus network contained edges with *p* ≤ 0.05 in four or five of the algorithms.

All ASVs in *G. barretti* were correlated (Spearman) with the signal of the diketopiperazines and tryptophan derivatives. In these two compound groups, the ASVs producing the ten strongest positive correlations were compared.

The online supplement including analysis code can be found at https://ksteffen.github.io/Paamiut/.

## Results

### The oceanographic setting revealed five water masses in the Davis Strait

**Figure 2:**
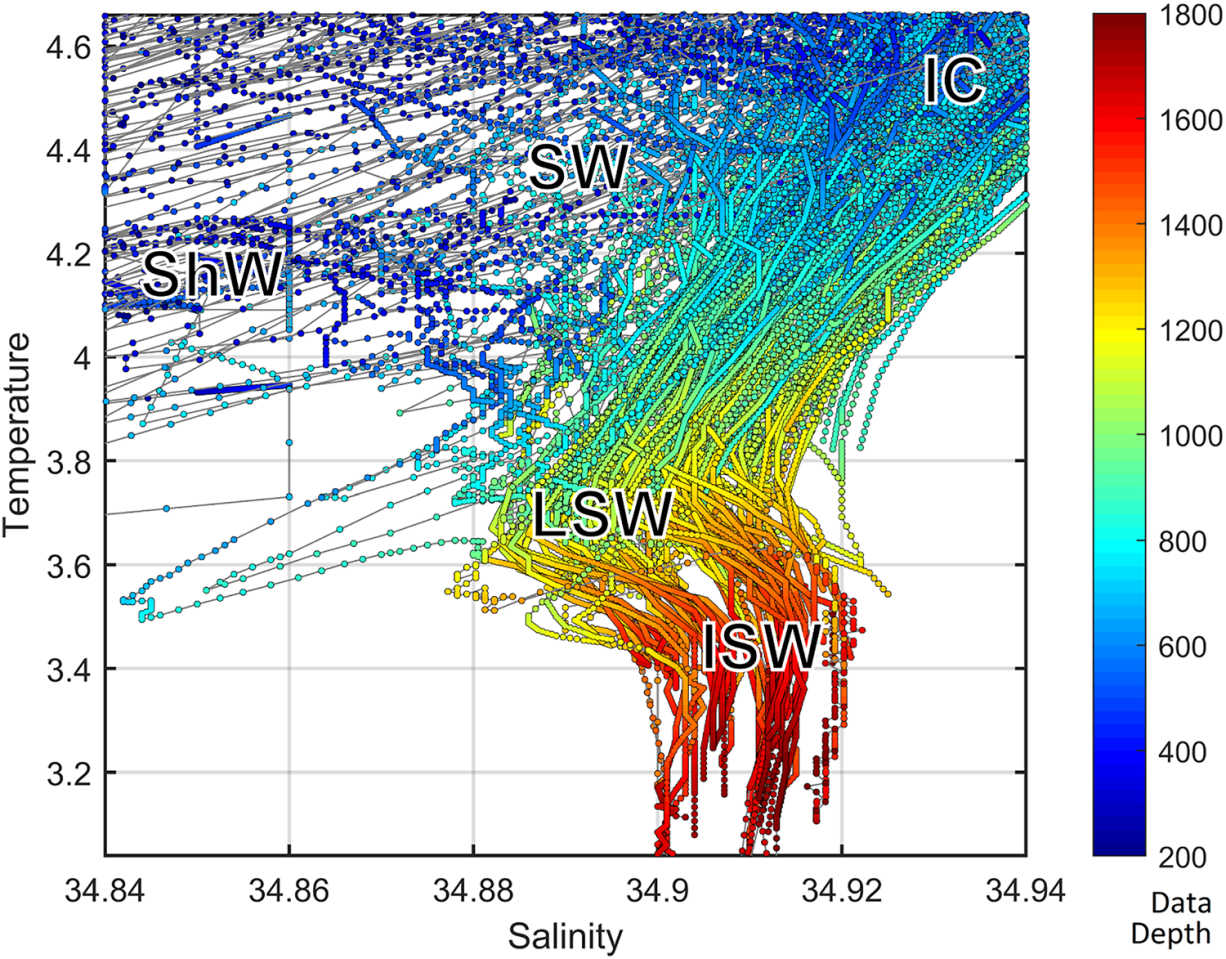
Temperature-salinity diagram showing the superimposed water masses at different depths in the Davis Strait. Shelf Water (ShW) and Slope Water (SW) are shallower than the samples in this study and display a range of salinities. We detect the core of the Irminger current (IC) at approx. 600 m depth, the core of the Labrador Sea Water (LSW) at approx. 1200 m depth, and the Icelandic Slope Water at approx. 1600 m and below. The abbreviations for the water masses were placed to approximate the cores of the water masses. Samples were attributed to the water masses based on their sample depth (see Table S1).

The Davis Strait where the samples were collected was found to be characterized by five water masses from shallow to deep: Shelf Water (ShW), Slope Water (SW), the Irminger Current (IC), Labrador Sea Water (LSW), and Icelandic Slope Water (ISW) (Fig. 2). The water masses can be detected by bends in the lines representing the continuous measurements of salinity and temperature with depth. Based on the sample depth, the samples in this study originated mainly from two water masses: those shallower than 1000 m were attributed to the IC with its core at about 600 m, a salinity of 34.93 psu, a temperature of 4.5 °C, and those from localities deeper than 1000 m were attributed to the LSW with its core at 1200 m, a salinity of 34.89 psu, a temperature of 3.7 °C. The limit of 1000 m depth was chosen to approximate the midpoint in depth between the two water masses. The LSW was more recently ventilated through winter convection in the Labrador Sea and therefore was richer in oxygen than the IC above it, which in turn was mainly advected from the North Atlantic outside the Labrador Sea. The strong currents intensify mixing between these two water masses and thus horizontal and vertical transfer of matter^52^. Even though the distinction between the water masses persists, this exchange may help transfer critical substances and enrich the water mass interfaces. The two shallowest specimens of *Weberella bursa* (224 and 331 m respectively) are influenced by the SW water mass in the Hudson Strait.

### Prokaryotic communities changed with depth

**Figure 3:**
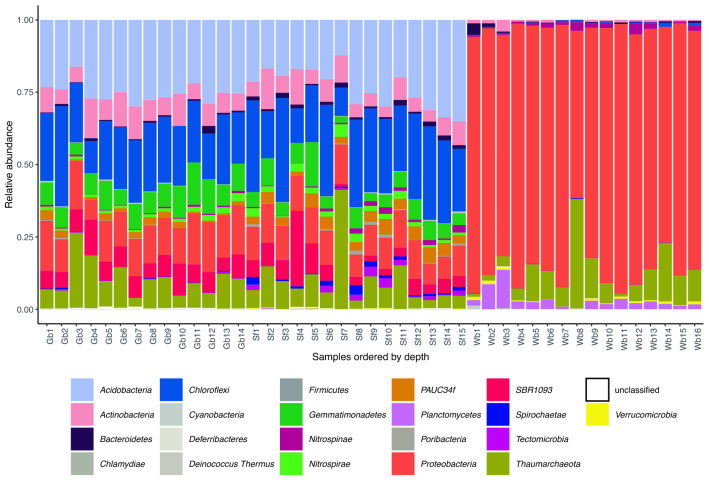
Prokaryotic community composition based on relative abundance of 16S rRNA gene sequences, aggregated at the phylum level. Specimens were grouped by sponge species and ordered by increasing depth from left to right, *Geodia barretti* (Gb, 407–1462 m), *Stryphnus fortis* (Sf, 483–1476 m) and *Weberella bursa* (Wb, 244–1271 m).

We recovered a total of 420 amplicon sequence variants (ASVs) belonging to 17 prokaryotic phyla associated to the 14 *G. barretti* specimens, 461 ASVs belonging to 20 prokaryotic phyla associated to the 15 *S. fortis* specimens, and 135 ASVs belonging to 13 prokaryotic phyla associated to 16 W*. bursa* specimens (Fig. 3). For individual specimens, the richness ranged from 148 to 229 (mean 192) ASVs for *G. barretti*, from 129 to 204 (mean 176) ASVs for S*. fortis*, and 16–58 (mean 29) ASVs for *W. bursa*. Although *S. fortis* is frequently overgrown with the sponge *Hexadella dedritifera*, we detected no signs of a contamination of the *S. fortis* microbiota as its composition is very different from that of *H. dedritifera* microbiota (Fig. S2 in^53^). The prokaryotic alpha diversity in *G. barretti* and *S. fortis* was higher than in *W. bursa* for all metrics assessed (Fig. S2)*.* This is evidence of *S. fortis* being an HMA sponge and *W. bursa* being an LMA sponge^54^. There were 98 (78.6% of the reads from *G. barretti*), 89 (77.6% of the reads from *S. fortis*), and 20 (94.8% of the reads from *W. bursa*) ASVs above the threshold of 0.25% average relative abundance. Among these abundant ASVs, 72 (73.5%), 64 (71.9%) and 11 (55%) were from sponge-enriched clusters (in *G. barretti*, *S. fortis*, and *W. bursa* respectively). As the prokaryotic communities were different for each sponge species (PERMANOVA of all 45 samples and all 691 ASVs by sponge species, *p* = 0.001), we performed further analyses per sponge species hereafter. The results of constrained and unconstrained analyses of environmental parameters and the prokaryotic communities, identified depth as significant factor for the microbiota composition, sometimes in combination with other environmental parameters (Table 1).

**Table 1:** Results for environmental analyses of the microbiota.

Unconstrained ordination					
Species	Environmental parameter	NMDS1	NMDS2	r^2^	*P* value
*G. barretti*	Depth	0.96701	− 0.25474	0.8945	0.001
*G. barretti*	Longitude	0.77996	0.62583	0.4251	0.047
*G. barretti*	Salinity	0.70615	− 0.70806	0.5171	0.025
*S. fortis*	Depth	0.84791	0.53014	0.6976	0.002
*S. fortis*	Longitude	0.68725	0.72642	0.6101	0.007
*W. bursa*	Depth	0.15403	0.98807	0.7720	0.001
*W. bursa*	Longitude	0.28260	0.95924	0.5937	0.006
*W. bursa*	Temperature	0.14099	0.99001	0.7231	0.003
*W. bursa*	Salinity	0.21497	0.97662	0.8490	0.001

Constrained ordination					
Species	Environmental parameter	Df	AIC	F	*P* value
**Stepwise model building using ordistep (CCA)**					
*G. barretti*	Depth	1	6.7097	3.4066	0.005
*S. fortis*	Depth	1	13.547	2.1462	0.005
*W. bursa*	Depth	1	25.701	1.6576	0.005
*W. bursa*	Latitude	1	25.993	1.4649	0.030

Constrained ordination					
Species	Df	Chi square	F	*P* value	
**ANOVA of the resulting models**					
*G. barretti*	1	0.34451	3.4066	0.001	
*S. fortis*	1	0.31199	2.1462	0.001	
*W. bursa*	2	0.8527	1.5888	0.001	

The upper section for unconstrained ordination allowed fitting of depth, latitude, longitude, salinity, temperature, sampling year at the same time.

Only significant (*p* < 0.05) factors are listed.

The lower section for constrained analyses included only environmental variables with a VIF < 10 to avoid multicollinearity.

For *G. barretti* and *W. bursa*, depth, latitude, temperature and sampling year were included, for *S. fortis* depth, latitude, sampling year and salinity (see Table S7 for VIFs).

**Figure 4:**
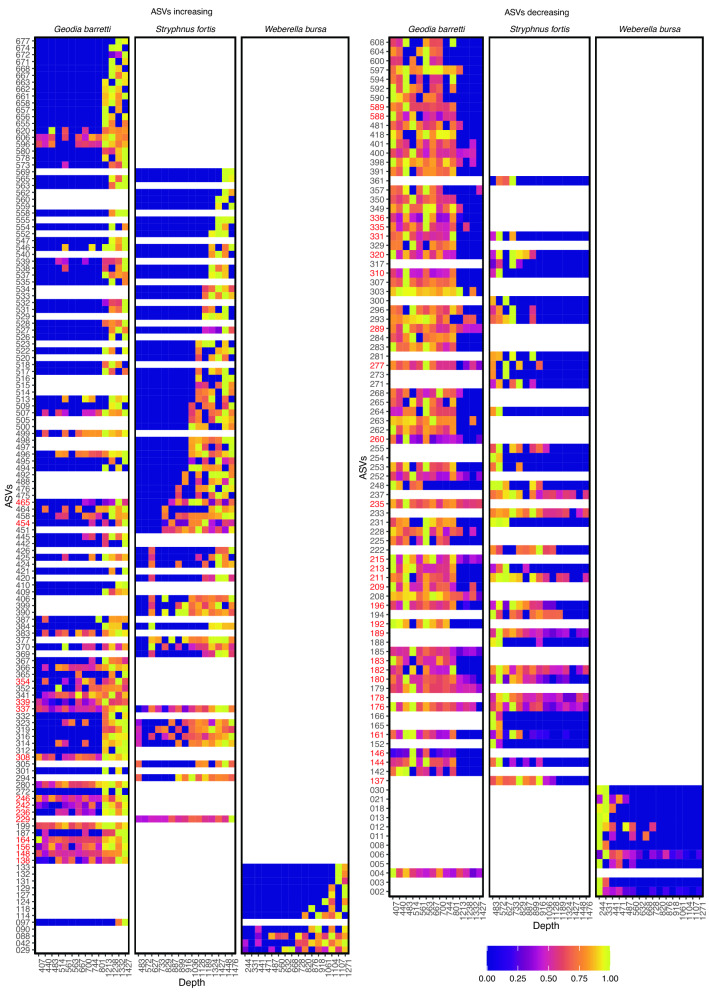
ASVs in *G. barretti*, *S. fortis* and *W. bursa* for which average relative abundance correlated with depth. The left panel shows ASVs increasing with depth, the right panel shows ASVs decreasing with depth in the three sponge respectively. ASV relative abundance is scaled from 0 to 1 for visualisation purposes. ASV labels coloured in red are sponge-enriched ASVs.

In *G. barretti*, 149 ASVs (35.5% of the ASVs representing an average relative abundance per sample of 45%) correlated with depth (86 increased, 63 decreased in relative abundance with increasing depth). In *S. fortis*, 99 ASVs (21.5% of the ASVs, average relative abundance per sample 21.2%) correlated with depth (62 increased, 37 decreased) and in *W. bursa*, 23 ASVs (17.0% of the ASVs, average relative abundance per sample 13.5%) correlated with depth (12 increased, 11 decreased) (Fig. 4). There was no apparent taxonomic trend or clustering among ASVs changing with depth. Hierarchical clustering of the prokaryotes revealed clusters of samples below 1000 m in *G. barretti* and *S. fortis* but not in *W. bursa* (Fig. S3). The sponge microbiota in the different water masses were significantly different in *G. barretti* and *S. fortis,* but not in *W. bursa* (PERMANOVA *p* = 0.004, *p* = 0.001, and *p* = 0.135 respectively, samples grouped above/below 1000 m, approximating the midpoint between the IC and LSW).

Sister group ASVs were defined as pairs of ASVs with sequence similarity ≥ 97% and opposing depth response. We found 19 pairs of sister group ASVs in *G. barretti*, two pairs in *S. fortis* and no instance of sister ASVs in *W. bursa*. These sister group ASVs included members of *Acidobacteria* (*Subgroups 6*, *9*, *15*, and *16*), *Proteobacteria* (*JTB23*), *Chloroflexi* (*SAR202 clade*, *TK10*, and *Anaerolineae*) and *Gemmatimonadetes* (*BD2-11 terrestrial group*) (Figs. S4, S5, Table S2).

### Metabolomes changed with depth

**Figure 5:**
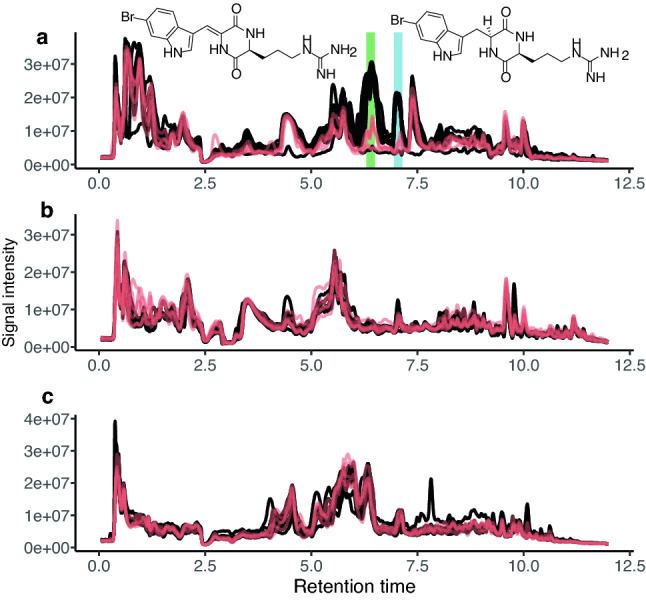
Overlain are the chromatograms acquired on a HILIC column (positive ESI) of (**a**) *G. barretti*, (**b**) *S. fortis*, and (**c**) *W. bursa*. Black lines indicate chromatograms from shallow specimens (< 1000 m), red lines indicate chromatograms from deep specimens (> 1000 m). For *G. barretti*, the green area highlights the peak of barettin (*E*- and *Z*-form), the blue area highlights the peak of 8,9-dihydrobarettin.

Metabolomes contained 3507, 2808, 4673, and 3166 features for HILIC positive, HILIC negative, RP positive and RP negative, respectively (“cleaned”). Further filtering to retain only entries identified as ions recovered 2212, 1351, 2736 and 1678 features respectively. Filtering to retain only the feature with the strongest signal per ‘pc_group’ (i.e., computationally approximating individual metabolites) recovered 105, 123, 171, and 105 features respectively. Overall, the metabolomes of the three holobionts were significantly different from each other (PERMANOVA, *p* = 0.001 in the HILIC positive “cleaned” data set) (Fig. S6). Therefore, we treated the metabolomes for the three species separately hereafter. Two major peaks containing barettin and 8,9-dihydrobarettin clearly decreased in deep specimens of *G. barretti* (Fig. 5). In contrast, the profiles for *S. fortis* and *W. bursa* did not show obvious variations in major peaks.

To evaluate whether depth affects the metabolomes as a whole, OPLS depth model fit (Table S3) was evaluated. Of the 36 depth models generated, 27 performed well (R^2^X > 0.9 and Q^2^ > 0.5), 11 of *G. barretti*, 9 of *S. fortis*, and 7 of *W. bursa*. Despite the many variables, cross validation indicated only six cases of overfitting (pQ2 > 0.05). The high number of good models is interpreted as support for the hypothesis that metabolome changed with depth.

**Figure 6:**
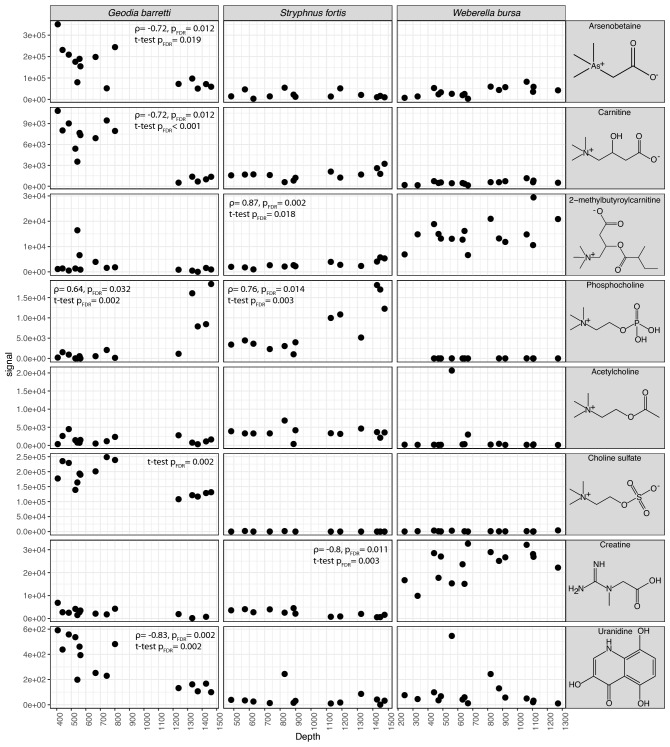
Signal intensities of annotated VIP compounds plotted against sample depth. Correlation tests and t-tests were performed to assess all compounds variation with depth. Significant results (*p*_FDR_ < 0.05) were annotated in the plot, correlation (ρ and *p*_FDR_) on top, and t-test (*p*_FDR_) below.

Since the HILIC positive data set contained the most steadily acquired data (Fig. S7), we extracted compound signals from it when possible. We identified VIPs (features with importance to the depth model) (Fig. 6), and confirmed their identity with reference compounds for arsenobetaine, carnitine, phosphocholine, acetylcholine, choline sulphate, creatine. For VIPs uranidine and 2-methylbutyroylcarnitin no reference compounds were available. Significant variation with depth was annotated in the corresponding plot, for the complete results see (Table S4). Acetylcholine is the only VIP not correlated with depth in any of the three species.

**Figure 7:**
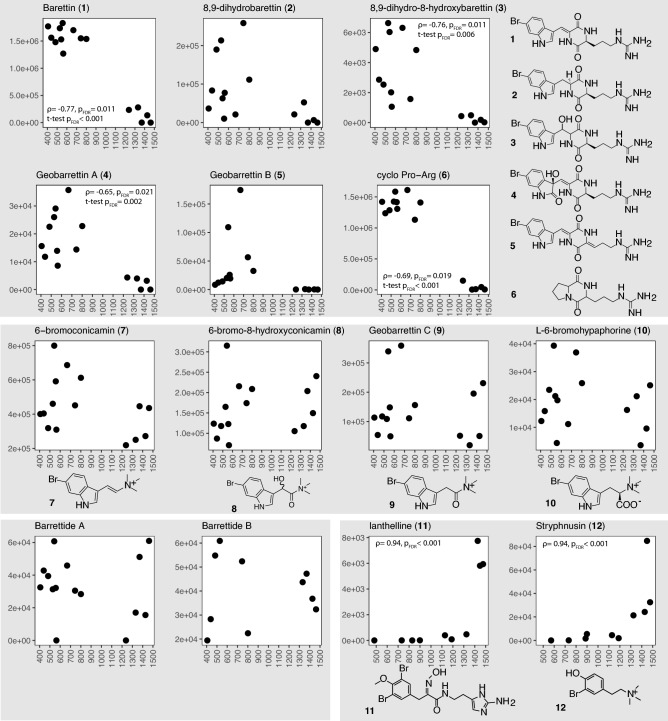
Signal intensities of previously known bioactive compounds plotted against sample depth. Correlation tests and t-tests were performed to assess all compounds variation with depth. Significant results (*p*_FDR_ < 0.05) were annotated in the plot, correlation (ρ and *p*_FDR_) on top, and t-test (*p*_FDR_) below. Compounds associated with *G. barretti*: top panel (**1**–**6**) are diketopiperazines, middle panel (**7**–**10**) are indole derivatives, bottom left are peptides. Compounds associated with *S. fortis*: bottom right panel (**11**, **12**).

In *G. barretti*, we identified 12 previously reported bioactive compounds diketopiperazines Trp-Arg (barettin^55^, 8,9-dihydrobarettin^56^, 8,9-dihydro-8-hydroxybarrettin^57^, geobarrettin A and B^58^), diketopiperazine Pro-Arg^57^, bromotryptophane derivatives (6-bromo-8-hydroxyconicamin^25^, 6-bromoconicamin^25^, geobarrettin C^58^, L-6-bromohypaphorine^58^), and the peptides barrettides A and B^59^. We could not find bromobenzisoxalone barettin^60^ (Fig. 7), possibly due to its very low concentration in the animal^61^ or production under specific circumstances only. In *S. fortis*, ianthelline and stryphnusin, two previously reported compounds were annotated (Fig. 7). Significant variation with depth was stated in the corresponding plot, for the complete results see (Table S4). There are no previously reported compounds in *W. bursa*.

### Inter-omics analyses and identification of microbiota-metabolite links

Directed analysis of the *G. barretti* ASVs and specialised metabolites hypothesised to be of prokaryotic origin (diketopiperazines, tryptophan derivatives), revealed that four bacteria yielded strong positive correlations with three of the six diketopiperazines: ASVs 144, 264, 284, all members of Acidobacteria and ASV 171, a member of Actinobacteria. Six prokaryotes yielded strong positive correlations with three of the four tryptophan derivatives: ASV 144, 283, 284 all members of Acidobacteria, 320 a member of Proteobacteria, 350 a member of Chloroflexi, and 307, an unclassified prokaryote. ASVs 144, 171, 283 and 320 were categorised as “common ASVs” and all ASVs with strong positive correlations are found both in *G. barretti* and *S. fortis*.

To visualise patterns among the *G. barretti* ASVs, we constructed a consensus network of positive microbial interactions, containing 117 nodes (ASVs) and 143 edges (Fig. S8A). ASVs mentioned previously as correlating with specialised metabolites were highlighted with bold read labels. ASVs in the network correlating with depth were mainly found in distinct clusters (Fig. S8B). Results for testing overall congruency or correlation between microbiota and metabolome matrices were inconclusive and can be found in the supplement (Tables S5, S6).

## Discussion

### Sponge-associated prokaryotic communities are influenced by the water mass

Sponge-associated prokaryotic communities varied across meso- and bathypelagic depths (Fig. 4), a pattern which was masked when ASVs were aggregated at phylum level (Fig. 3). This finding contradicted the generally perceived stability of the sponge microbiome^62–66^ which changes mainly under lethal stress or disease^67^. The extent of ASV change was more pronounced in HMA than LMA sponges, but we were able to detect it in both types. For *G. barretti*, we also found a significant effect of longitude and salinity on the associated prokaryotic community (Table 1), but the variance inflation factors indicate that caution must be taken in interpreting these environmental variables due to their collinearity with depth (Table S7). For the *S. fortis* prokaryotic community composition, longitude may play an additional role (if only in the form of distance-decay). For *W. bursa*, the impact of environmental variables on prokaryotic community composition were inconclusive because they varied with the method used.

We conceptualized depth as a proxy for other environmental (biotic/abiotic) factors, as depth per se is unlikely to affect the sponge or its prokaryotic community. Oceanic depth correlates with light availability, temperature, salinity, and hydrostatic pressure, yet none of these parameters are likely to affect the samples in this study. Light intensity was ruled out as influencing factor in this study as even the shallowest sponge samples originated from localities beyond the euphotic zone and cyanobacteria were mostly absent^68^. Further, neither temperature^62,69^ nor salinity^64^ on their own necessarily affect sponge prokaryotic communities in shallow water except under fatal temperature stress conditions^70^. Hydrostatic pressure affects enzyme activity and thereby metabolic rates in fish in abyssal and hadal depths^71^. While these depths are beyond our sample range, the effect of hydrostatic pressure on the holobionts in this study could not be ruled out. Two previous studies investigating the overall effect of depth found little to no evidence for an effect on sponge prokaryotic communities^9,65^. This was likely due to the sampling within the same water masses and low number of samples in general.

Additional conceivable parameters for explaining the shifts in prokaryotic communities would be nutrients, minerals, dissolved oxygen and carbon dioxide, but the number of in situ measurements for these were insufficient to support quantitative conclusions. However, many of these parameters are dependent on the oceanographic setting, i.e. water masses. In context of geographical position and depth, temperature and salinity are known to sufficiently resolve the water mass structure, ventilation history and mixing conditions^34,72,73^. In particular, it has been demonstrated that the LSW formed by winter convection in the Labrador Sea is much better ventilated (higher dissolved oxygen and carbon dioxide) and is more recent in formation (younger in age) than the Atlantic Water that is advected to the region with the IC. We thus attributed the change of prokaryotic communities to variations in the vertical water mass structure and characteristics as detected by our oceanographic analyses. Since the dynamics and oceanographic structure of the water column changed in transition from the sea interior onto the shelf, water depth could be used as a variable effectively representing the cross-slope changes in seawater and ecosystem characteristics at the bottom level and throughout the water column.

In the HMA sponges *G. barretti* and *S. fortis*, where the effect of depth on prokaryotic community composition was most pronounced, we found samples below 1000 m forming distinct clusters (Fig. S3). This grouping was supported by the approximate position of the oceanic front between the two water masses: the IC and the LSW. This indicated the shallower sponge specimens (< 1000 m depth) were mainly influenced by the warmer and saltier IC (core at approx. 500 m depth). Meanwhile the deeper specimens (> 1000 m depth) were mainly influenced by the colder and fresher LSW (core at approx. 1200 m depth).

This grouping was further supported by the observation that differences in prokaryotic communities in deep and shallow sponges were sometimes caused by substitutions of taxonomically close ASVs (“sister group ASVs”) potentially representing microbial ecotypes of two different water masses. We reported such a pattern mainly among the *G. barretti* prokaryotes. However, in terms of the number of such ecotype pairs recovered, we found their detection to be strongly influenced by the stringency of the significance threshold (*p* < 0.05 versus *p*_FDR_ < 0.05) and detection strategy (correlation test or t-test for depth response) highlighting the difficulty of assessing the phenomenon. Generally, less stringent thresholds revealed more pairs of sister group ASVs and additional phyla (Table S3, Figs. S4, S5).

Previous findings support the hypothesis of the effect of water masses on the microbiota, as water masses are known to structure microbial distributions in the sea^74,75^. They act as dispersal barrier for microorganisms in the deep sea, and each water mass harbours specific prokaryotic communities with different ecotypes^76–79^. Even communities of unicellular eukaryotes^80,81^, fish or benthic invertebrates seem structured by water masses^82–86^. Recent work linking clusters of sponge microbiota to a seamount could likewise be interpreted as sign of the effect of water masses and currents around the structure^10^.

In addition to this general effect of water masses on marine life, we acknowledge that population structure in the sponge hosts themselves could be a factor contributing to the shifts observed in the prokaryotic community composition of the three sponges. It is conceivable that water masses stratify dispersal of sponge larvae thereby leading to isolated populations^86,87^. However, as a part of the sponge prokaryotic community would still be derived from the different water masses, sponge-host population structure is not mutually exclusive with the hypothesis proposed herein.

Taken together, the present study has shown for the first time that water masses have a major impact in the structuring of sponge-associated prokaryotic communities, and thereby could explain the prokaryotic community variation observed in the three deep-sea sponge species.

Given the resilience of the prokaryotic taxa associated with sponges, even upon disturbance^63,64^, the sponge microbiome appears set once fully acquired. It is further recognized that the microbiota composition is dictated mainly by the sponge host identity^2,9,12^.

This poses the question as to how and when the adult sponge microbiota is established. Despite ample evidence for vertical inheritance of microbes^1,3,4^, its consistency in terms of which taxa are passed on has been challenged recently^6^ giving more weight to environmental acquisition. The pattern described herein, i.e. i) the compositional stability at phylum level despite ii) variations of underlying ASVs could be interpreted as evidence for a selective process for the required taxa, where representation of the taxa at ASV strain level depends on the availability in the environment. The presence of corresponding ASV ecotypes in samples from different water masses suggests a functional and taxonomic redundancy or complementarity^88,89^. Therefore, we hypothesize these taxa to participate in equivalent functions in the holobiont.

### The chemical profiles of sponge holobionts vary with depth

Thus far, factors affecting specialized metabolite production in sponges have often been inconclusive or contradictory^26,27,90,91^, e.g. regarding seasonality^28,92^ or reproduction^26,27,93^. In the present study, evidence for variation of the chemical profiles with depth could be found at all levels of the analyses, with the most pronounced effects in the HMA sponges. The multivariate analyses attributed a proportion of the variation of all three holobiont metabolomes to depth and these models in turn aided in the identification of specific features varying with depth. Signals of previously known metabolites (Fig. 7) as well as additionally reported compounds (Fig. 6) extracted from the chemical profiles further confirmed variations with depth.

Among the VIP compounds creatine, carnitine and choline sulphate, are known osmolytes^94,95^ also called osmoprotectants or compatible solutes, regulating the osmolarity of the cell^94^. Arsenobetaine^96^ and acetylcholine^95^ and phosphocholine (and choline) are suggested to be involved or precursor to betaine synthesis, another osmolyte. The compound 2-methylbutyroylcarnitine^97^ was found to vary in intertidal mussels (Fig. S9, Table S4 for extended results) and thus could also be an osmolyte. The variation of osmolyte levels seems logical as salinity of the surrounding water varies with depth (Fig. 2). Although quantitative comparisons cannot be made based on our data, it appears that the major osmolytes differed in each holobiont, as did their respective variation with depth.

Ianthelline (a bromotyrosine derivative) and stryphnusin (a bromophenetylamine derivate) were originally isolated from verongiid sponges and are hence likely to be produced by the encrusting verongiid *Hexadella dedritifera* commonly overgrowing *S. fortis*^98^. As we found that most of the samples of *S. fortis* had traces of overgrowth, we hypothesize that *H. dedritifera* produces more of the compounds with depth (Fig. 7). Although ianthelline production may vary^98^, the clear increase with depth is an unexpected finding. In the shallow water verongiid *Aplysina aerophoba,* bromotyrosine derivative variations are correlated with water temperature and season^92^, whereas our results suggested other factors associated with depth. The signals of bromotryptophane derivatives and barrettides A and B, as well as C^99^ in *G. barretti* remained unaffected by depth.

### Bioactivity perspective

In general, diketopiperazines (DKPs) are implicated in bacterial communication as quorum sensing molecules^100^. *G. barretti*-associated DKPs (Fig. 7 compounds 1–6) changed strongly in signal intensity across the different depth which would seem illogical under the assumption that quorum sensing was their only function. Indeed, several other bioactivities have been assayed for the barettins (all DKPs except for Pro-Arg). Barettin and 8,9-dihydrobarettin are antifouling^56^, selective 5-HT serotonin receptor ligands^60^ and acetylcholine esterase inhibitors^25^; and geobarrettin B has anti-inflammatory properties^58^. Interestingly, similar activities have been reported for the tryptophan derivatives (Fig. 7 compounds 7–10). 6-bromoconicamin inhibits acetylcholine esterase^25^, geobarrettin C has anti-inflammatory properties^58^ and L-6-bromohypaphorine is an agonist of α7 nicotinic acetylcholine receptor^101^. It is difficult to disentangle complex interactions e.g. when hypothesising about producers of these compounds among the rich and diverse sponge microbiota but from ranking the correlation analysis in these two compound groups, we found members of Acidobacteria accounted for half the ASV top hits. There are no cultured members from sponges and little is known about their lifestyle, but they are found globally as a dominant group in HMA sponges^102^. Although only few classes of Acidobacteria have been investigated for their functional potential it is suggested that their genomes frequently encode a large number of specialized metabolites^103,104^.

However, questions remain as all the ASV top hits were shared by *G. barretti* and *S. fortis*, and yet the barettins are characteristic to the former only. Among the ASV top hits, the abundant ASVs 144 and 171 present at high relative abundance are more likely to produce a compound as abundant as barettin (compared to low abundance ASVs 264 and 284 present at low relative abundance).

## Conclusions

This study showed that depth impacted prokaryotic communities and metabolites associated with sponge holobionts. Water masses, as approximated by depth, are known to structure marine life and were shown here to stratify holobiont microbiota alike. For both HMA sponges, but especially *G. barretti*, substitutions of distinct pairs of highly related ASVs present in deeper or shallower specimens, respectively, suggested that a part of the functional potential of the holobionts is preserved despite ASV replacement. Although sponge-associated prokaryotes and metabolites covary with depth, it is challenging to link specific metabolites to specific prokaryotes (or the sponge itself) without additional orthogonal data or study design. Correlating ASV abundance with metabolite signals showed an overrepresentation of members of Acidobacteria among the results. From a bioprospecting and chemical ecology point of view, our finding that sponge samples from different water masses harboured unique ASVs and displayed increase and decrease of bioactive compounds further suggests that the deep-sea and its inhabitants are an untapped source for novel compounds, even for species that have already been screened in shallower waters.

## Supplementary Information

Supplementary Information.

Supplementary Table S1.

Supplementary Table S2.

Supplementary Table S3.

Supplementary Table S4.

Supplementary Table S5.

Supplementary Table S6.

Supplementary Table S7.

Supplementary Table S8.

Supplementary Table S9.

Supplementary Table S10.

Supplementary Table S11.

Supplementary Table S12.

Supplementary Table S13.

Supplementary Table S14.

## Data Availability

Sequencing data was deposited at the NCBI Sequence Read Archive (SRA) with accession number SRP142603 under file numbers SRX3993926–SRX3993970 (https://www.ncbi.nlm.nih.gov/bioproject/PRJNA453542). UPLC-HRMS data was deposited at MetaboLights (www.ebi.ac.uk/metabolights/MTBLS1388). Extended methods and data sets were documented in the supplementary information for this article. Analysis code can be found at https://ksteffen.github.io/Paamiut/.
