# Supplementary material for: Oceanographic setting influences the prokaryotic community and metabolome in deep-sea sponges
Source: Sci Rep. 2022 Mar 1;12:3356. doi: 10.1038/s41598-022-07292-3 (PMC8888554; doi:10.1038/s41598-022-07292-3)
Supplement: Supplementary file 1 — Supplementary Information. [file 41598_2022_7292_MOESM1_ESM.pdf]

Supplementary Information

Oceanographic setting influences the prokaryotic community and metabolome in deep-sea sponges

Karin Steffen, Anak Agung Gede Indraningrat, Ida Erngren, Jakob Haglöf, Leontine E. Becking, Hauke Smidt, Igor Yashayaev, Ellen Kenchington, Curt Pettersson, Paco Cárdenas & Detmer Sipkema.

1. Overview

Here, we reported water masses as approximated by water depth to influence the prokaryotic community composition and holobiont metabolome in three deep-sea sponge species, *Geodia barretti*, *Stryphnus fortis* and *Weberella bursa*. By 16S rRNA gene sequencing and computational statistical analyses, we assessed and analysed the operational taxonomic units (ASVs) that increased or decreased with depth and found distinct groups of ASVs in samples associated with two water masses (Labrador Sea Water and Irminger Current) in the Davis Strait, North Atlantic. In parallel, by Ultra-high Performance Liquid Chromatography coupled with High-Resolution Mass Spectrometry (UPLC-HRMS) we acquired and analysed the metabolome of these sponge holobionts. Using computational analyses, we found that the metabolomes changed with depth. We extracted and identified metabolite signals driving the depth-dependent changes as well as signals of known bioactive compounds. Finally, we combined microbiota and metabolome data to generate a hypothesis about the producer of the antibiofounling compound barettin. This multi-omics data acquisition and analysis approach relied on the use of several instruments and methods that are detailed in the experimental section. The supplementary methods described herein apply to the wet lab procedures. The code and comments for the subsequent computational analyses were deposited at the Paamiut GitHub repository.

The large amount of data produced in this study had several additional aspects and implications which were presented and discussed in the supplementary results section.

EXPERIMENTAL

Additional sampling notes

During the sampling on the Pâmiut cruises, the sponges remained on deck or in the laboratory for approximately 30–45 min before they were frozen to -20 °C. The outside temperature usually oscillated around 4–5 °C, and the sorting area was approximately 10 °C. The sample consisted of both choanosome and coretx.

2. UPLC-HRMS extended methods

Laboratory methods and data acquisition

All samples were processed in randomized order throughout extraction and analyses using glass instruments during the extraction to avoid chemical contamination. Metabolites were separated in downstream mass spectrometry (HRMS) analysis using two different chromatographic columns (UPLC): a hydrophilic interaction liquid chromatography (HILIC) column that favours retention of polar compounds, and a reversed-phase (RP) column that favours retention of non-polar compounds.

Mass spectrometry analysis

Dried extracts were dissolved in 200 μL solvent (HILIC: 50 μL H_2O and 175 μL MeCN; RP: 140 μL H_2O and 10 μL MeCN) in glass vials. Upon addition of the organic solvent for HILIC chromatography, all samples separated into two immiscible layers. The vials were centrifuged for 3 min at 2000 x g to yield an even separation. Only the top layer (approximately 150 μL) was transferred to a Chromacol 03-FISV MS-vial (Thermo Scientific, Waltham, Massachusetts, USA) for MS analyses. For RP chromatography, no layers were observed, and the entire volume of the dissolved sample was used. A five μL aliquot from each individual MS-vial for HILIC and RP, respectively, was combined to produce a quality control (QC) sample.

High resolution MS analysis system and settings

The extracts were analyzed back-to-back in positive and negative ionization mode on an Acquity I-Class Ultra Performance Liquid Chromatography (UPLC) coupled to a G2S Synapt Q-TOF with an electrospray ionization (ESI) ion source (all Waters Corp., Milford, MA, USA). Chromatographic separation in HILIC mode was performed on an Acquity UPLC BEH Amide column (1.7 μm, 2.1 mm inner diameter × 50 mm, Waters Corp.). Mobile phase A consisted of 95:5 MeCN/MQ water with 5 mM ammonium formate and 0.1

% formic acid (FA), and mobile phase B consisted of 40:60 MeCN/MQ water with 5 mM ammonium formate and 0.1 % FA. The gradient elution profile was as follows: mobile phase A was decreased non-linearly (slope factor 8, MassLynx) from 100 % A to 100 % B over 14 min, 100 % B was held for 2 min and then decreased back to 100 % A over 1 min. The column was re-equilibrated at 100 % A for 6 min for a total runtime of 23 min. Chromatographic separation in RP was performed on an Acquity UPLC BEH C18 column (1.7 μ m, 2.1 mm inner diameter × 50 mm, Waters Corp.). Mobile phase A consisted of MQ water with 0.1 % FA, and mobile phase B was MeCN with 0.1 % FA. The gradient elution profile started at 95 % A, was decreased linearly over 14 min to 5 % A, and 5 % A was held for 2 min before the column was re-equilibrated at 95 % A for 4 min. The flow rate was set to 0.4 mL/min, the column temperature was set to 40°C, the samples were kept at 8°C and the injection volume was 5 μL in all experiments.

Data acquisition

Data acquisition was performed using MS^E mode, and lock mass correction was applied using a solution of leucine enkephalin in both positive and negative mode. Ionization parameters were set as follows in positive/negative mode; the capillary voltage was 1kV/1.5 kV, the cone voltage was 30 V/25 V, the source offset was 50/60 and the source temperature was set to 120 °C. Nitrogen was used as desolvation and cone gas with gas flows of 800 L/h and 50 L/h, respectively, and desolvation temperature was set to 500 °C/450 °C. For MS^E acquisition a collision energy ramp from 20–45 eV was used with argon as collision gas. The instrument was calibrated in the *m/z* range 50–1500 using sodium formate prior to each analysis. All study samples were analysed in both RP and HILIC, in positive and negative ionization mode, resulting in four metabolite datasets per sponge specimen. The column and sample cone were cleaned in between each analysis mode. Prior to each analysis 10 QC injections were made to condition the column, and to ensure stable retention times and signal intensities. The study samples were analysed in randomized order with QC injections interspaced every sixth injection.

MS data processing

Raw files were converted to netCDF files by Databridge (part of MassLynx, Waters Corporation, Milford, Massachusetts, USA). The netCDF files with the chromatographic spectra were sorted into folders according to species and processed with XCMS in R. Peak picking was performed using the centWave function with parameters ppm=8, peakwidth set to c(5,45) and the noise parameter set to 2000. Retention time alignment was performed with the obiwarp function and the response factor set to 10, grouping was performed with the “group” function and the “fillPeaks” function was used to impute a signal in cases where no matching

pseudospectra were detected. The data set was curated to remove features eluting in the void (retention time less than 45 s). A raw data set as well as two normalized data sets (Log10-transformed and median fold change normalized) were produced and filtered to only retain features with a coefficient of variation $< 30\%$ in the QC samples. After subsequent evaluation, raw (i.e. untransformed) data sets were used in subsequent statistics and modelling.

Peak picking with XCMS and annotation with CAMERA

We processed samples from all three sponnge species in random order with interspersed injection of a combined QC sample to monitor stability of the UPLC-HRMS run. The acquired signals/spectra were converted to netCDF format using the Program DataBridge, and thereafter sorted into four folders, three for the sponge species (Gb, Sf, Wb) and one for the QC samples (QC). Peak picking and combination of pseudospectra is performed with the R package xcms, the subsequent annotation of adducts and isotopes with the R package CAMERA. Code for XCMS and CAMERA data processing is found at the Paamiut GitHub repository

3. 16S gene sequencing extended methods

DNA was extracted from sponge samples with the FastDNA Spin Kit for soil (MP biomedicals, Santa Ana, CA, USA) following manufacturer's instructions including two bead beating steps for 45 s (Precellys 24 Bertin Instruments, Montigny-le-Bretonneux, France). DNA concentrations were measured using a spectrophotometer (DeNovix DS-11, Wilmington, NC, USA) and its size was visualised on a 1% agarose gel. The V4 region of the 16S rRNA gene was amplified with the EMP (Earth Microbiome Project) primer pair 515FY and 806RB [1], [2]. The PCR reactions contained 16.55 μL nuclease-free water (Promega, Madison, USA), 5 μL of 5× HF buffer, 0.2 μL of 2 U/μL Phusion hot start II high fidelity polymerase (Thermo Fisher Scientific, Waltham, MA, USA), 0.75 μL of 10 μM stock solutions of each primer, 0.75 μL 10 mM dNTPs (Promega, Madison, WI, USA) and 1 μL template DNA (10 – 20 ng/μL) for a total volume of 25 μL. Amplification was performed with an initial denaturation at 98°C for 3 min, 25 cycles at 98°C for 25 s, 50 °C for 20 s, 72 °C for 20 s and a final extension of 7 min at 72 °C. PCR products were visualised on a 1% (w/v) agarose gel. Five μL of the PCR products were used as template in the second PCR reaction to incorporate eight nucleotide sample-specific barcodes, as previously described [3]. This second PCR was performed in triplicate for each sample. The PCR reactions contained 31 μL nuclease-free water (Promega), 10 μL of 5× HF buffer, 0.5 μL of 2 U/μL Phusion hot start II high fidelity polymerase (Thermo Fisher Scientific), 5 μL equimolar mixes of 10 μM forward primer (barcode-linker-Unitag1) and reverse primer

(barcode-linker-Unitag2), 1 μL 10 mM dNTPs (Promega) and 2.5 μL of the first PCR product as template for a total of 50 μL.

Annotation of SpongeEMP according to Dat *et al.*

The sequences of the abundant ASVs for each sponge species (relative abundance > 0.25%) were subjected to a BLAST search against a curated sponge Earth Microbiome Project (EMP) database (https://github.com/amnona/SpongeEMP). The sponge microbiome project subASV sequences that were identical or had one nucleotide mismatch with sequences of the most abundant ASVs were uploaded to the spongeEMP online server (www.spongeemp.com) to identify ASVs that were significantly enriched in sponges [4].

RESULTS & DISCUSSION

4. Metabolomics: OPLS models

OPLS models are prone to overfitting when there are more variables than samples. This premise is met by our data sets that have vastly more variables (metabolomic features) than sponge samples. Therefore, in order to validate the model, a sevenfold cross validation is performed in the process of generating it, i.e. when calling the `opls()` function. The predictive power of a model is denoted Q2. For the actual data model it is "Q2 cum", and for the cross-validation models with permutated data it is "pQ2". In case of overfitting models (effectively modelling random noise), the predictive power of the cross-validated models should be similar or higher. In our case, in Tab. S3, we see that in two models (*S. fortis*: RP neg: ion, and *G. barretti*: RP neg: ion) the predictive power of permutated data is higher than the original data. There are few additional cases where models on permutated data performed similar to original data (*S. fortis*: HILIC pos: ion/RP pos: ion, *W. bursa*: RP pos: cleaned/pc group). However, there is no exact cut-off to define overfitting. We conclude thus that for most of the models presented in the table, the models seem to model data and not noise and that our conclusions that depth affects the metabolome are valid.

Metabolomics: Choline and serotonin/histamine

Biofouling inhibition is an activity tested and shown for several of the compounds from *G. barretti* and *S. fortis* belonging to different compound classes (diketopiperazines, peptides, bromotyrosin-derivatives [5], [6]. Two hypothetic modes of action have been proposed for the biofouling inhibition of barettin and 8,9-dihydrobarettin [7]. One of these acts via 5-HT serotonin receptors as especially barettin has a high affinity

to some of the receptor subtypes [8] which are also present in barnacles. The other mode involves inhibition of acetylcholinesterase (AChE), as barettin, 8,9-dihydrobarettin and stryphnusin inhibit the enzyme at concentrations close to other inhibitors of marine origin [7], [9]. AChE in turn was found in larval organs involved in obtaining settlement cues [10].

Given the implicated effect on choline; and the hypothesised connection with serotonin, we isolated their signals in the metabolomes and correlated them with depth. Serotonin was absent or present in very low relative abundance in *G. barretti*, present in low amounts in *W. bursa* and in low to moderate relative abundance in *S. fortis*, whereas choline was abundant in all three sponges (Fig. S9). Serotonin (ρ=0.58, p=0.04) and choline (ρ=0.72, p=0.0075) increased with depth in *S. fortis*. (Fig. S9). Serotonin [11], [12] and serotonin-derived compounds [13] have been previously identified with NMR in demosponges but genes coding for serotonin receptors and serotonin-synthesizing enzymes have so far not been found in sponge genomes [14]. The near absence of serotonin in *G. barretti* and the possible absence of serotonin receptors in sponges suggest that barettin targets are more likely to be macro- or microfoulers with serotonin-like receptors, and not the sponge itself, but its presence in the other sponges does not rule out the possibility entirely. As serotonin is a neurotransmitter, we further searched for histamine (Fig. S9). The signal intensity of histamine did not correlate with depth in any of the sponges.

Metabolome: uranidine

One VIP compound that was tentatively identified as Uranidine, a quinolone yellow pigment, originally isolated from the yellow shallow water sponge *Aplysina aerophoba* [15]. Uranidine was shown to have anti-HIV activity [16]. The compound has never been recorded outside of the verongiid sponges and, if it is confirmed, this is only the third record of this compound, this time in the deep-sea sponge *G. barretti*. We should stress that this compound was identified based on *m/z* 194.0448 [M+H]^+ and fragments (MSMS), without a reference. Uranidine is known to be an unstable compound that oxidises easily to a blue quinone that polymerizes and turns black [15]. On damaged areas of *G. barretti*, purple spots can be observed on the usually whitish surface and whitish to peach coloured choanosome (P. Cárdenas, *pers. obs.*). The reasons for the decrease of this compound with depth is currently unclear.

Metabolome: osmolytes

The role and function of the poorly understood arsenobetaine in marine environments has been investigated previously [17]. Arsenate might be taken up indiscriminately from the marine environment through

phosphate membrane transport systems of microbes. Due to the structural similarity with betaine, another osmolytes, it has been hypothesised to have the same function. However, the signals of both compounds in the three different sponges as well as their depth responses did not warrant unequivocal conclusions (Fig. S9). In *G. barretti* arsenobetaine decreased with depth (r=-0.705, p= 0.003) while betaine increased (r= 0.589, p= 0.021). In *S. fortis*, the signal remained stable and in *W. bursa*, arsenobetain increased with depth (r= 0.625, p= 0.01). The opposing responses to depth might be due to differences in the holobionts' depth response. However, if indiscriminately taken up by the environment, it would be expected that the signals of betaine and arsenobetaine would correlate. This was only the case in *W. bursa* (r= 0.631, p= 0.009).

5. Microbiome

Thaumarchaeota are one of the important constituents of sponge-associated prokaryotic communities, associated with nitrogen cycling, and particularly ammonia oxidation [18], [19]. ASV146 and ASV138 have only one bp difference, ASV138 is identical (140 bp query coverage) to a Thaumarchaeota clone which belongs to a sponge-enriched clade including *Cenarchaeum symbiosum* [20]; this clone was present in the sponges *G. barretti* and *Phakellia ventilabrum* (Bergen area, Norway, 200-300 m) (e.g. JQ612474), as well as *Stelletta normani* KF597128 (Porcupine Bank, 1350 m). ASV146, showed generally higher relative abundances in specimens collected above 1000 m, but was absent in specimens collected below 1000 m, in both *G. barretti* (Fig. 4) and *S. fortis* (average relative abundance; shallow Gb 0.20 and Sf 0.14 versus deep Gb 0 and Sf 0.004). Conversely, ASV138 relative abundance is significantly increasing with depth, and is more abundant below 1000 m , but only in *G. barretti* (average relative abundance; shallow Gb 0.21 and Sf 0.16 versus deep Gb 0.55 and Sf 0.02) (Fig. 4). The inverse relationship of the relative abundance of the two crenarchaeal ASVs 138 and 146 with depth indicates that they are "sister-strains" and may represent two ecotypes from the two water masses: an IC-derived sponge-specific ecotype and a deeper LSW-derived sponge-specific ecotype. In a similar way, the Thaumarchaeota ASV004 (genus *Candidatus* Nitrosopumulis) has several sister-strains with opposite trends. ASV004 abundance is decreasing with depth in *G. barretti*, while ASV097 (1 bp difference) increase with depth in *G. barretti.* The fact that ASV004 matches the sequence of the surface water mass Thaumarchaeota ecotype [20] (16S sequence courtesy of O. Müller) suggests that these sponge ASVs are this time seawater derived and may reflect the water mass Thaumarchaeota community [20]. Hence, both sponge-enriched and seawater-derived ASVs are related to the water masses, and lead to similar overall phylogenetic composition of prokaryotic communities at the species level.

Data supplied for reproducibility

In order to make sure our research is reproducible, we provide the ASV table (Tab. S8), the ASV taxonomy (Tab. S9), the initial "cleaned" metabolome data sets for HILIC positive, HILIC negative, RP positive, RP negative respectively (Tab. S10, S11, S12, S13) and the metabolite signal intensities (Tab. S14).

Supplementary figures

All figures were produced with R v 3.5.1. A full list of packages and their versions is documented in the electronic supplementary Paamiut GitHub repository.

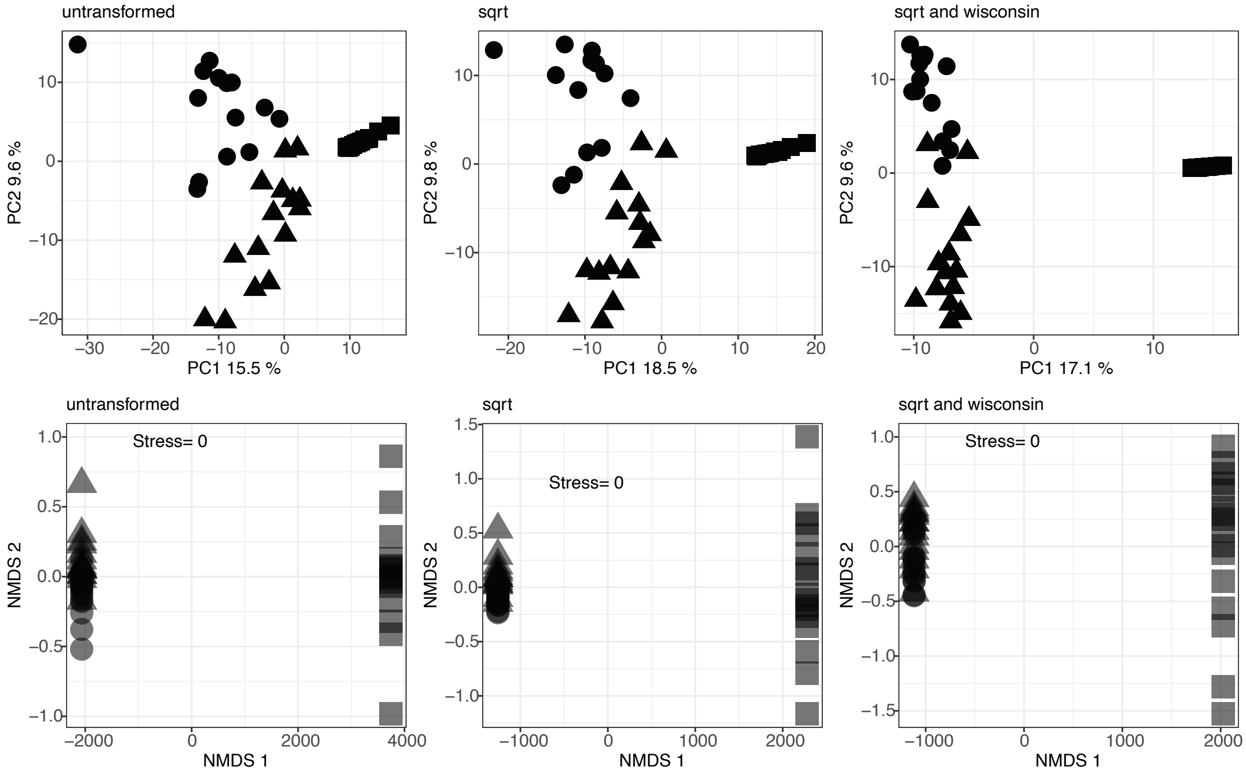

Figure S1: PCA (upper row) and NMDS (lower row) of the three sponge microbiota, and the effect of different data transformations. Circles are *G. barretti*, triangles are *S. fortis*, and squares are *W. bursa* samples.

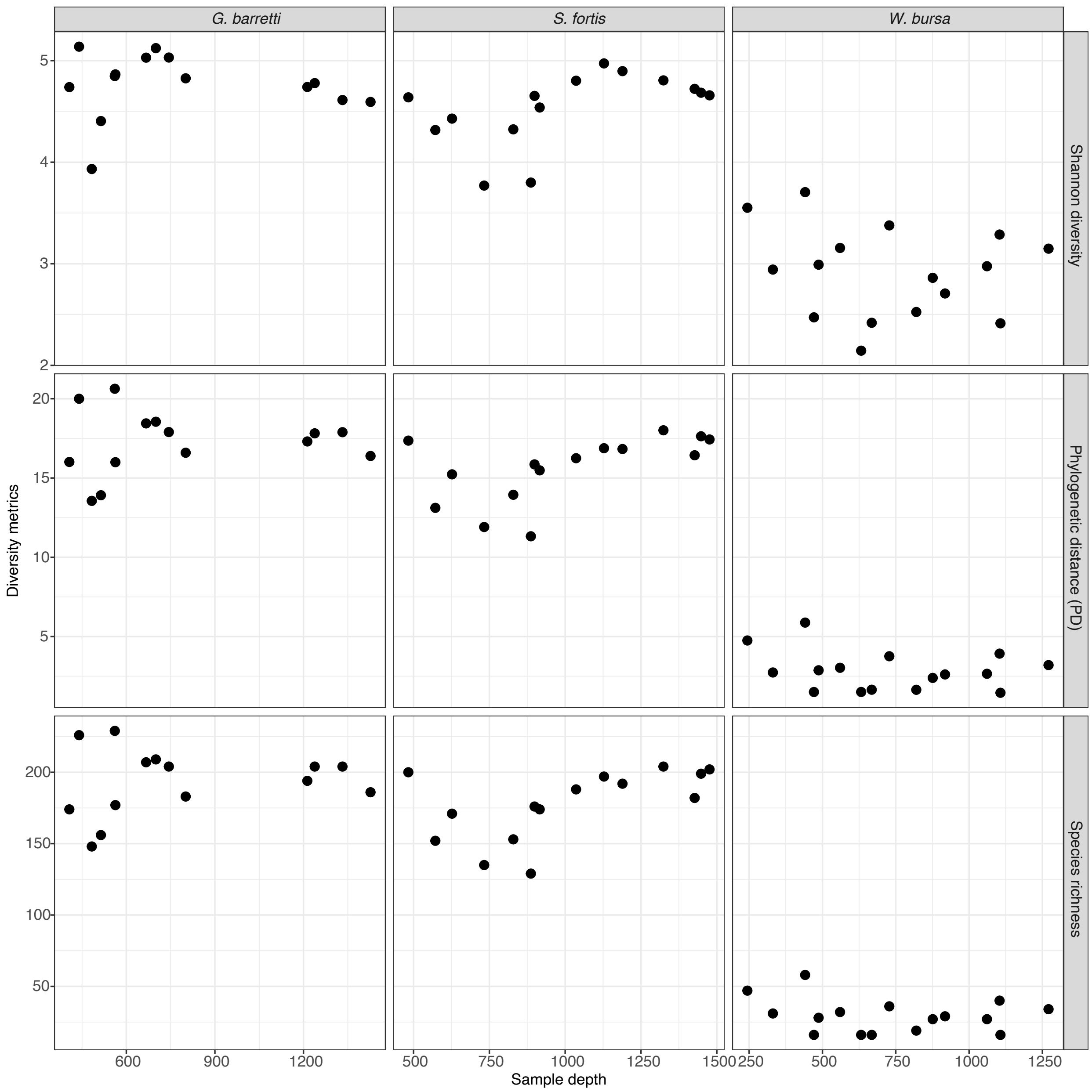

Figure S2: Alpha diversity of the specimens as measured by Faith's phylogenetic distance, species diversity and species richness. The diversity indices are higher for both HMA sponges (*G. barretti* and *S. fortis*) than for the LMA sponge *W. bursa*.

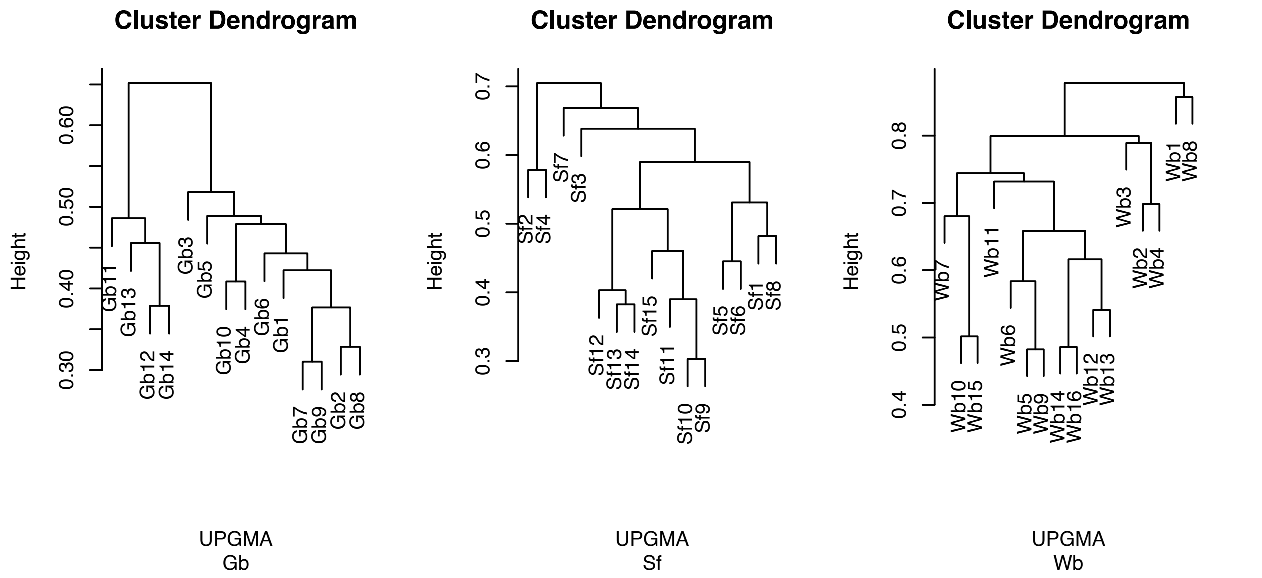

Figure S3: Hierarchical clustering of the ASVs in the three sponges revealed clusters of deep samples (>1000 m) in *G. barretti* (Gb11-Gb14) and *S. fortis* (Sf9-Sf15). No such clustering was evident in *W. bursa.* Figures were produced with R v. 3.5.1 and the vegan package.

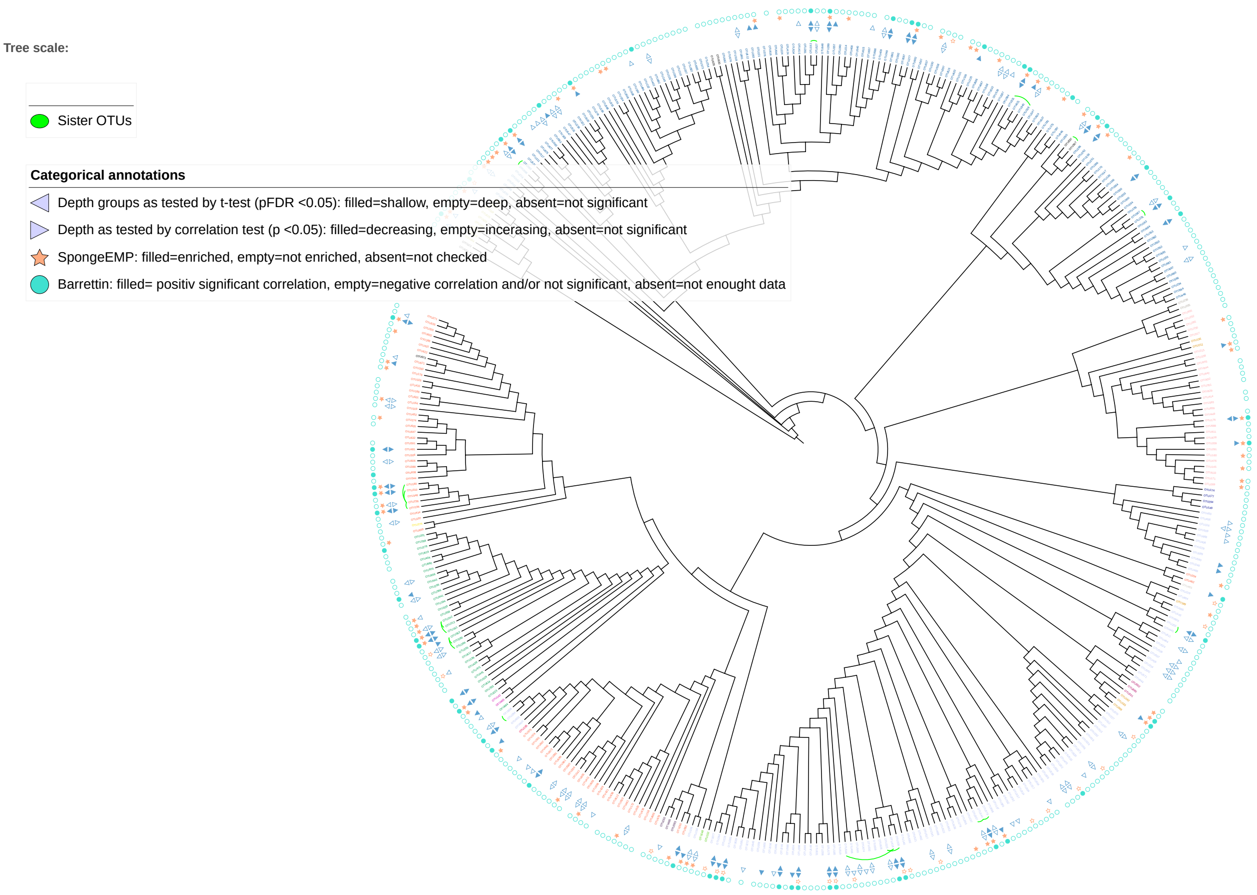

Figure S4: Annotated cladogram of all ASVs in *G. barretti*. Green arches indicate sister ASVs (n=19) as defined by t-test (pFDR<0.5) comparing relative abundance above and below 1000 m. The figure was produced with iTol and deposited online iToL (https://itol.embl.de/shared/karin_steffen).

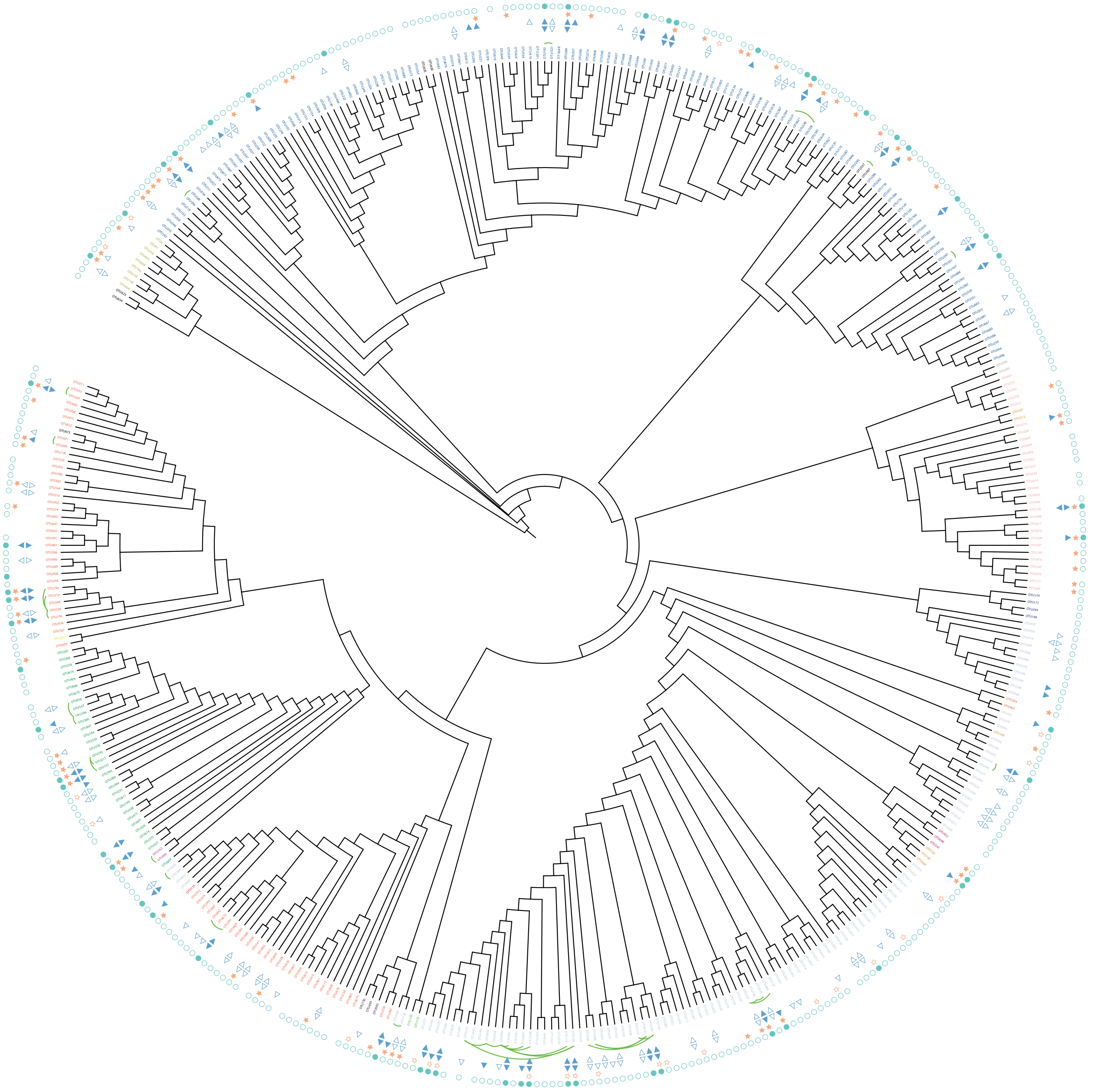

Figure S5: Annotated cladogram of all ASVs in *G. barretti*. Green arches indicate sister ASVs (n=35) as defined by correlation test (p<0.5) of relative abundance with depth. The figure was produced with iTol and deposited online iToL (https://itol.embl.de/shared/karin_steffen).

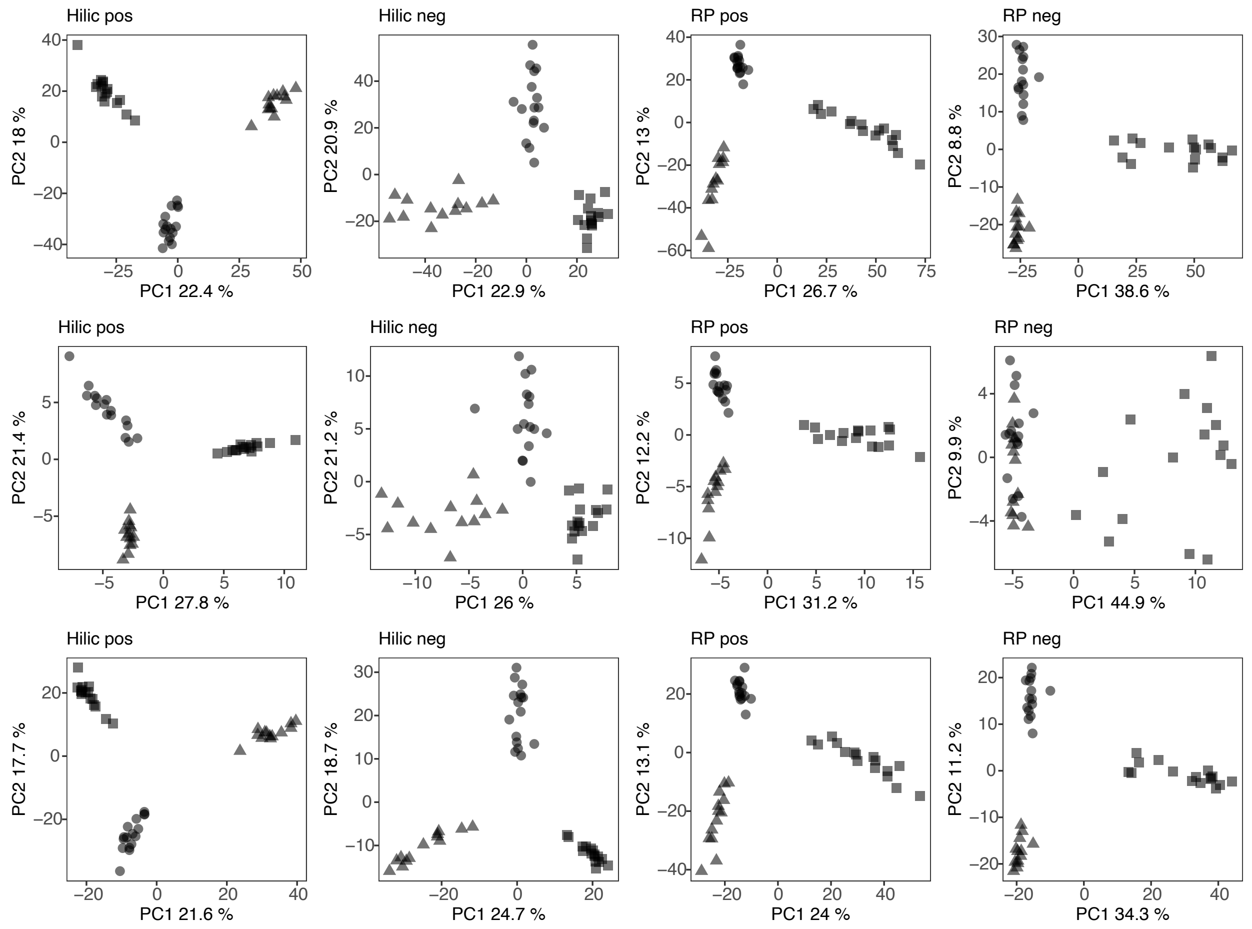

Figure S6: PCA of the metabolomes upon different filtering options. Top row: “clean” filtered data sets with 3 507, 2 808, 4 673, and 3 166 features for HILIC positive, HILIC negative, RP positive and RP negative, respectively. Middle row: “ion” filtered data sets with 2 212, 1 351, 2 736, and 1 678 features respectively. Bottom row: “pc_group” filtered with 105, 123, 171, and 105 features respectively. Dots are *G. barretti* samples, triangles are *S. fortis* samples, and squares are *W. bursa* samples.

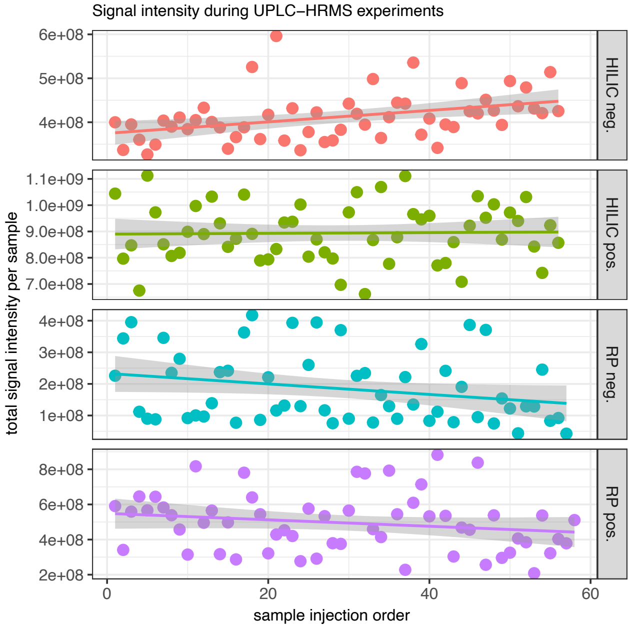

Figure S7: Total HRMS signal intensity per sample in the order of their injection in the instrument. The HRMS signal is most stable for HILIC positive UPLC.

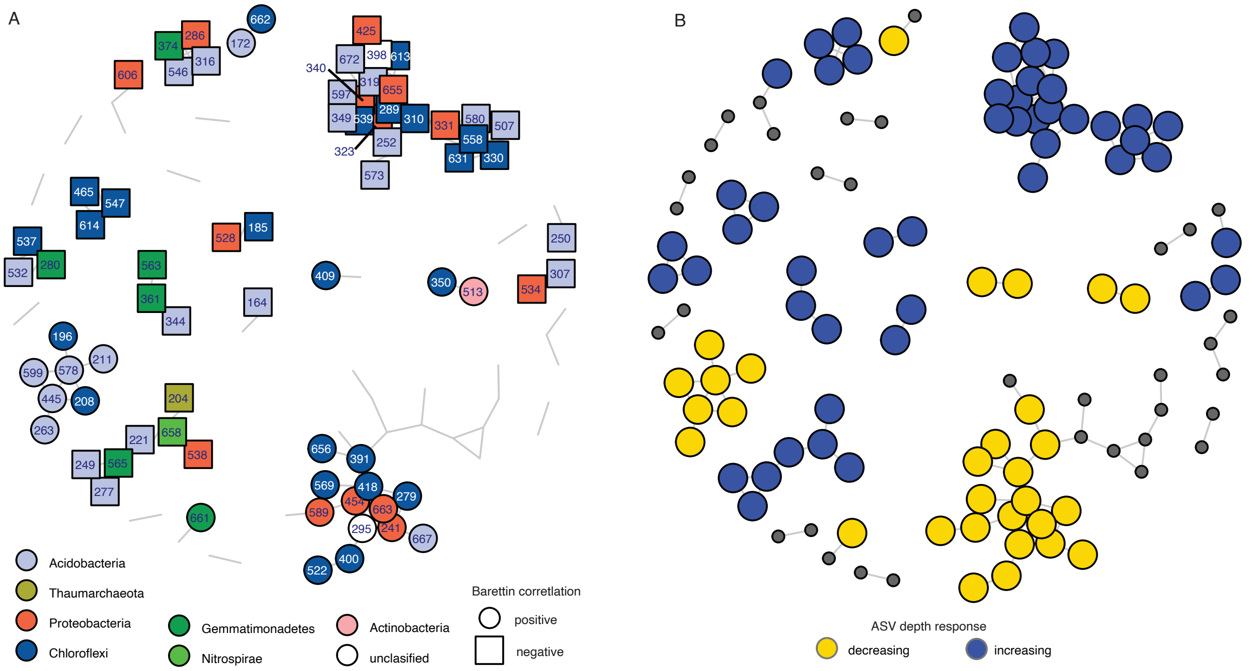

Figure S8: A consensus microbial interaction network of ASVs in *G. barretti* present in more than three samples (n=289) was generated based on MENA, MIC, SparCC and fastLSA. The network contained 117 nodes (ASVs) and 143 edges between them and is annotated with (A) barettin correlation and (B) depth response of the ASVs. We hypothesised that a compound as abundant as barettin could influence the prokaryotic community within the host, i.e. stimulate certain ASVs (circles in A) or suppress (squares in A). Of the six shortlisted ASVs, three are found in the network. However, as barettin and depth were strongly correlated, the clusters of ASVs also matched their depth response (B) and we cannot separate the two factors. The figure was produced in R v. 3.5.1 with package igraph.

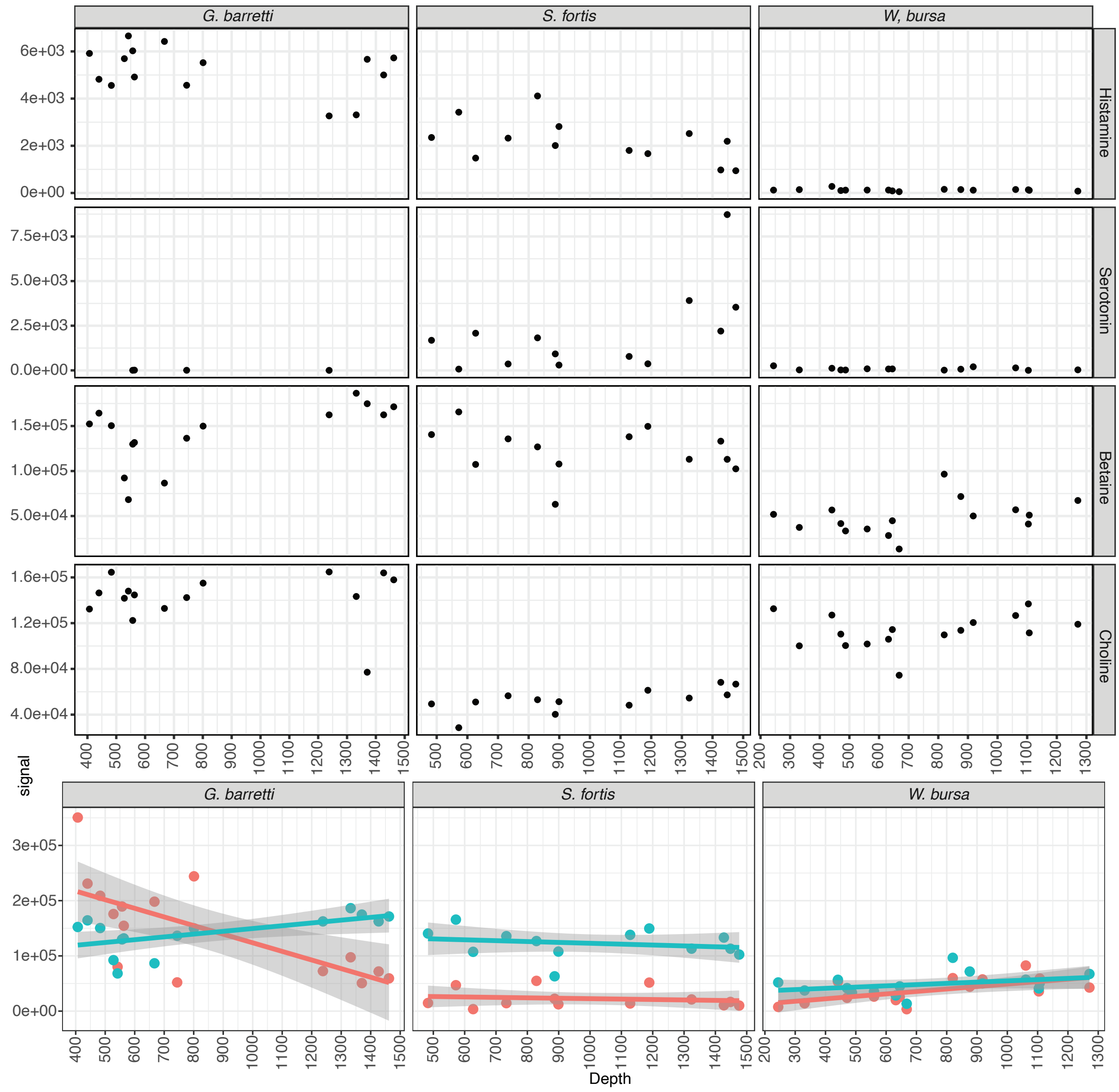

Figure S9: Additonal compounds signals extracted from the metabolome. Upper panel: As actelycholine was designated a VIP, we searched for the presence of additional mammalian neurotransmitters, finding histamine and serotonin. Likewise, after finding several osmoprotectants among the VIPs we targeted choline and betaine as both compounds are also implicated in osmoregulation. Lower panel: signal intensities of arsenobetaine (red) and betaine (turquoise) and a linear regression for each series.

Supplementary tables

- **Tab. S1**: Pangaea sample metadata, i.e. Col_no = collection number, Mission = Paamiut mission identifier, Set, Species, Depth = sample depth, Latitude, Longitude, MeanBottomTemp_Cdeg = Temperature °C in situ, MeanBotSalinity_PSU = salinity in situ, GEAR = sampling gear, YEAR = sampling year, DATE), unified_ID. The unified_ID was used throughout the manuscript and data analyses as sponge sample identifier, and links these samples with the metabolomic (LC-MS HILIC positive, LC-MS HILIC negative, LC-MS RP positive, LC-MS RP negative) and sequencing data (NCBI SRA#). This file was called Steffen_et_al_metadata_PANGAEA.csv in the online analysis code supplement.

- **Tab. S2**: Sister ASVs: A table containing pairs of ASVs (ASV1 and ASV2) with sequence similarity > 97% (exact value given in "pairwise sequence similarity") and opposing response to depth as indicated in column "evaluation". Only ASVs within the same sponge species were compared.

- **Tab. S3**: Diagnostics of the 36 OPLS models based on all combinations of experimental setups (HILIC pos/neg, RP pos/neg, n=4), species (n=3), and feature filtering ("cleaned, "pc_group", "ion"; n=3)). A pQ2 >0.05 is indicative of overfitting and the 6 models where that was the case are thus not supported.

- **Tab. S4**: Correlations and t-test of all compounds (Fig. 6, 7) with depth in all three sponges.

- **Tab. S5**: Mantel test results (Mantel statistic, p-values) for the comparison of congruent microbiota and metabolome data sets.

- **Tab. S6**: Procrustes and protest results (Procrustes sum of squares, correlations in symmetric rotation, p-values) for the comparison of congruent microbiota and metabolome data sets.

- **Tab. S7**: Variance inflation factors (VIF) for building models in constrained ordination. "Full model"(*) was the maximum number of parameters included to avoid multicollinearity. These parameters and the resulting model were used for the 'ordistep' function. For inclusion, none of the parameters can have a VIF > 10.

Table S7: Stepwise elimination of the environmental parameter with the highest VIF lead to the maximum inclusive "full" model denoted with an * for the ordistep function.

Species	Depth	Latitude	Longitude	Temperature	Salinity	Year	X
G. barretti	5.61	8.52	13.30	31.12	51.53	2.29	
G. barretti	5.12	6.41	13.15	2.06		2.27	
G. barretti	1.23	1.78		1.93		1.65	*
S. fortis	10.90	6.56	5.52	26.68	24.59	1.42	
S. fortis	4.22	5.08	5.07		3.65	1.08	*
W. bursa	5.79	10.53	13.72	17.68	29.37	1.30	
W. bursa	4.83	7.76	13.68	2.48		1.25	
W. bursa	2.09	1.24		2.37		1.07	*

- **Tab. S8**: ASV table. This file was called ASV_all_R.csv in the online analysis code supplement. Compared to the naming of ASVs in the manuscript, these original data sets (Tab. S8, S9) contained a leading serial number "196900" that was removed for convenience in the manuscript.

- **Tab. S9**: ASV taxonomy. This file was called microbiome_taxonomy.csv in the online analysis code supplement. Compared to the naming of ASVs in the manuscript, these original data sets (Tab. S8, S9) contained a leading serial number "196900" that was removed for convenience in the manuscript.

- **Tab. S10**: Table S10 "HILIC positive cleaned" is part of the four metabolome data sets that can be used to reproduce all general metabolome analyses outlined in the online analysis code supplement, HILIC_pos_20190417_cleaned.csv.

- **Tab. S11**: Table S11 "HILIC negative cleaned" is part of the four metabolome data sets that can be used to reproduce all general metabolome analyses outlined in the online analysis code supplement, HILIC_neg_20190421_cleaned.csv.

- **Tab. S12**: Table S12 "RP positive cleaned" is part of the four metabolome data sets that can be used to reproduce all general metabolome analyses outlined in the online analysis code supplement, RP_pos_20190421_cleaned.csv.

- **Tab. S13**: Table S13 "RP negative cleaned" is part of the four metabolome data sets that can be used to reproduce all general metabolome analyses outlined in the online analysis code supplement,

RP_neg_20190422_cleaned.csv.

- **Tab. S14**: Metabolites signal intensities. These signals intensities were manually extracted, annotated or identified from the chromatograms. When comparing the same compound across samples, these values can be interpreted as relative quantities. This file is called metabolite_master_20190605.csv in the online analysis code supplement. The metabolite names are abbreviated: GeoA = Geobarrettin A, GeoAi = Geobarrettin A isomer, GeoB = Geobarrettin B, GeoC = Geobarrettin C, L6bhp = L-6-bromohypaphorine, dhhb = 8,9-dihydro-8-hydroxybarettin, choline, chol_s = choline sulphate, bet = betaine, mbc = 2-methylbutyroylcarnitine, cre = creatine, brc = 6-bromoconicamin, acl = acetylcholine, asb = arsenobetaine, bar = barettin, crn = carnitine, cp4 = 6-bromo-8-hydroxy-conicamin, dhb= 8,9-dihydrobarettin, ian = ianthelline, pch = phosphocholine, sol = cyclo Pro-Arg, sfn = stryphnusin, ura = uranidine, ser = serotonin, his = histamine.

References

[1] A. Apprill, S. McNally, R. Parsons, and L. Weber, "Minor revision to V4 region SSU rRNA 806R gene primer greatly increases detection of SAR11 bacterioplankton," *Aquatic Microbial Ecology*, vol. 75, no. 2, pp. 129–137, Jun. 2015, doi: 10.3354/ame01753.

[2] A. E. Parada, D. M. Needham, and J. A. Fuhrman, "Every base matters: Assessing small subunit rRNA primers for marine microbiomes with mock communities, time series and global field samples: Primers for marine microbiome studies," *Environmental Microbiology*, vol. 18, no. 5, pp. 1403–1414, May 2016, doi: 10.1111/1462-2920.13023.

[3] H. J. van Lingen *et al.*, "Diurnal Dynamics of Gaseous and Dissolved Metabolites and Microbiota Composition in the Bovine Rumen," *Frontiers in Microbiology*, vol. 8, Mar. 2017, doi: 10.3389/fmicb.2017.00425.

[4] T. T. H. Dat, G. Steinert, N. Thi Kim Cuc, H. Smidt, and D. Sipkema, "Archaeal and bacterial diversity and community composition from 18 phylogenetically divergent sponge species in Vietnam," *PeerJ*, vol. 6, p. e4970, Jun. 2018, doi: 10.7717/peerj.4970.

[5] S. Ortlepp *et al.*, "Antifouling Activity of Bromotyrosine-Derived Sponge Metabolites and Synthetic Analogues," *Marine Biotechnology*, vol. 9, no. 6, pp. 776–785, Nov. 2007, doi: 10.1007/s10126-007-9029-x.

[6] K. Ø. Hanssen *et al.*, "The Bromotyrosine Derivative Ianthelline Isolated from the Arctic Marine Sponge Stryphnus fortis Inhibits Marine Micro- and Macrobiofouling," *Marine Biotechnology*, vol. 16, no. 6, pp. 684–694, 2014, doi: 10.1007/s10126-014-9583-y.

[7] E. K. Olsen *et al.*, “Marine AChE inhibitors isolated from *Geodia barretti*: Natural compounds and their synthetic analogs,” *Organic & Biomolecular Chemistry*, vol. 14, pp. 1629–1640, 2016, doi: 10.1039/C5OB02416A.

[8] E. Hedner *et al.*, “Brominated Cyclodipeptides from the Marine Sponge *Geodia barretti* as Selective 5-HT Ligands,” *Journal of Natural Products*, vol. 69, no. 10, pp. 1421–1424, 2006, doi: 10.1021/np0601760.

[9] L. W. K. Moodie *et al.*, “Synthetic analogs of stryphnusin isolated from the marine sponge Stryphnus fortis inhibit acetylcholinesterase with no effect on muscle function or neuromuscular transmission,” *Organic & Biomolecular Chemistry*, vol. 14, no. 47, pp. 11220–11229, 2016, doi: 10.1039/C6OB02120D.

[10] A. S. Clare, R. K. Freet, and M. McClary, “On the antennular secretion of the cyprid of *Balanus amphitrite amphitrite* , and its role as a settlement pheromone,” *Journal of the Marine Biological Association of the United Kingdom*, vol. 74, no. 1, pp. 243–250, Feb. 1994, doi: 10.1017/S0025315400035803.

[11] A. Guerriero, M. D’Ambrosio, F. Pietra, C. Debitus, and O. Ribes, “Pteridines, Sterols, and Indole Derivatives from the Lithistid Sponge Corallistes undulatus of the Coral Sea,” *Journal of Natural Products*, vol. 56, no. 11, pp. 1962–1970, Nov. 1993, doi: 10.1021/np50101a015.

[12] H. L. Castellanos, W. H. Mayorga, and C. B. Duque, “Estudio de la composición química y actividad antifouling del extracto de la esponja marina *Cliona delitrix*,” vol. 17, no. 2, pp. 209–224, 2010.

[13] M. Salmoun, C. Devijver, D. Daloze, J.-C. Braekman, and R. W. M. van Soest, “5-Hydroxytryptamine-Derived Alkaloids from Two Marine Sponges of the Genus Hyrtios,” *Journal of Natural Products*, vol. 65, no. 8, pp. 1173–1176, Aug. 2002, doi: 10.1021/np020009+.

[14] S. P. Leys, J. L. Mah, P. R. McGill, L. Hamonic, F. C. De Leo, and A. S. Kahn, “Sponge Behavior and the Chemical Basis of Responses: A Post-Genomic View,” *Integrative and Comparative Biology*, vol. 59, no. 4, pp. 751–764, Oct. 2019, doi: 10.1093/icb/icz122.

[15] G. Cimino, S. De Rosa, S. De Stefano, A. Spinella, and G. Sodano, “The zoochrome of the sponge verongia aerophoba (‘Uranidine’),” *Tetrahedron Letters*, vol. 25, no. 27, pp. 2925–2928, Jan. 1984, doi: 10.1016/S0040-4039(01)81328-9.

[16] S. Loya, A. Rudi, R. Tal, Y. Kashman, Y. Loya, and A. Hizi, “3,5,8-Trihydroxy-4-quinolone, a Novel Natural Inhibitor of the Reverse Transcriptases of Human Immunodeficiency Viruses Type 1 and Type 2,” *Archives of Biochemistry and Biophysics*, vol. 309, no. 2, pp. 315–322, Mar. 1994, doi: 10.1006/abbi.1994.1119.

[17] A. Popowich, Q. Zhang, and X. C. Le, “Arsenobetaine: The ongoing mystery,” *National Science Review*,

vol. 3, no. 4, pp. 451–458, Dec. 2016, doi: 10.1093/nsr/nww061.

[18] R. Radax, F. Hoffmann, H. T. Rapp, S. Leininger, and C. Schleper, “Ammonia-oxidizing archaea as main drivers of nitrification in cold-water sponges,” *Environmental Microbiology*, vol. 14, no. 4, pp. 909–923, 2012, Available: http://dx.doi.org/10.1111/j.1462-2920.2011.02661.x

[19] F. Zhang, L. Pita, P. M. Erwin, S. Abaid, S. López-Legentil, and R. T. Hill, “Symbiotic archaea in marine sponges show stability and host specificity in community structure and ammonia oxidation functionality,” *FEMS Microbiology Ecology*, vol. 90, no. 3, pp. 699–707, Dec. 2014, doi: 10.1111/1574-6941.12427.

[20] O. Müller *et al.*, “Spatiotemporal Dynamics of Ammonia-Oxidizing Thaumarchaeota in Distinct Arctic Water Masses,” *Frontiers in Microbiology*, vol. 9, pp. 1–13, 2018, doi: 10.3389/fmicb.2018.00024.
